# Nasal microbiota profiles in shelter dogs with dermatological conditions carrying methicillin-resistant and methicillin-sensitive *Staphylococcus* species

**DOI:** 10.1038/s41598-023-31385-2

**Published:** 2023-03-24

**Authors:** Sara Horsman, Erika Meler, Deirdre Mikkelsen, John Mallyon, Hong Yao, Ricardo J. Soares Magalhães, Justine S. Gibson

**Affiliations:** 1 School of Veterinary Science, The University of Queensland, Gatton, QLD 4343 Australia; 2 School of Agriculture and Food Sciences, The University of Queensland, St Lucia, QLD 4072 Australia; 3 Centre for Nutrition and Food Sciences, Queensland Alliance for Agriculture and Food Innovation, The University of Queensland, St Lucia, QLD 4072 Australia; 4 Children Health and Environment Program, Child Health Research Centre, The University of Queensland, South Brisbane, QLD 4101 Australia

**Keywords:** Antimicrobials, Bacteria, Microbial communities

## Abstract

Dermatological conditions may be complicated by *Staphylococcus* spp. infections influencing skin and nasal microbiota. We investigated the associations between the resident nasal microbiota of shelter dogs with and without dermatological conditions carrying methicillin-resistant and -sensitive *Staphylococcus* spp. Nasal sampling of 16 dogs with and 52 without dermatological conditions were performed upon shelter admission (baseline), and then bi-weekly until discharge (follow-up). All samples were cultured for *Staphylococcus* spp., while 52 samples underwent microbiota analysis*.* Two elastic net logistic regression (ENR) models (Model 1—baseline samples; Model 2—follow-up samples) were developed to identify predictive associations between dermatological conditions and the variables: signalment, antimicrobial treatment, and nasal microbial genera. Follow-up nasal samples of dogs with dermatological conditions had decreased microbiota diversity and abundance compared to dogs without dermatological conditions. Our ENR models identified predictive differences in signalment and nasal microbial genera between baseline and follow-up samples. Co-occurrence networks showed nasal microbial genera were more dissimilar when comparing dogs with and without dermatological conditions at follow-up. Overall, this study is the first to investigate *Staphylococcus* spp. carriage effects on nasal microbial genera in a canine animal shelter population, and ultimately reveals the importance of investigating decolonisation and probiotic therapies for restoring nasal microbiota.

## Introduction

Dogs admitted to animal shelters often arrive with dermatological conditions caused by infectious agents including mites and dermatophytes, and allergens associated with fleas, food, and the environment^1^. Dogs with these conditions often have secondary bacterial infections predominately caused by *Staphylococcus* spp., particularly *Staphylococcus pseudintermedius*^2,3^.

*Staphylococcus pseudintermedius* forms part of the skin and nasal resident microbiota of healthy dogs^2,3^. As such, it is important to investigate the carriage of methicillin-resistant *Staphylococcus* (MRS) spp., as infection with this bacterium may further complicate treatment outcomes^4^. The repeated prescription of antimicrobials and longer treatment duration to treat secondary bacterial infections increases the risk of developing antimicrobial resistance, particularly methicillin resistance^5^.

Previous studies have reported the carriage of methicillin-resistant and -sensitive *S*. *pseudintermedius* (MRSP and MSSP, respectively), and methicillin-resistant and -sensitive *S*. *aureus* (MRSA and MSSA, respectively), from the nares, mouth, and/or perineum in healthy dogs attending veterinary clinics and in shelter dogs^6–10^. Studies have also isolated *S*. *pseudintermedius* from up to 92% of canine pyoderma cases^11–14^, with 12.7% to 43.1% being methicillin-resistant^12–15^. *S*. *pseudintermedius* was isolated from 20 to 95% of canine otitis externa cases^16–20^ and of these, 8.7% to 50% were methicillin-resistant^17,19,20^. Prior bacterial infections have been identified as a risk factor for MRSP nasal carriage in dogs^21^. Additionally, nasal MRSA-colonising isolates from children with atopic dermatitis (AD) have been linked to MRSA-infecting isolates in children with concurrent skin and soft tissue infections and AD^22^. Hence, understanding the role of MRS and methicillin-sensitive *Staphylococcus* (MSS) spp. nasal carriage in dogs with dermatological conditions is vital in deciding whether decolonisation therapies are required to decrease secondary infection risk^23^.

Few publications to date have investigated the microbiota of dogs with skin allergies^24–27^. Using 16S rRNA gene amplicon sequencing, it has been reported that the nares of allergic dogs had lower species richness compared to healthy dogs^24^. The predominant genera in the nares of allergic dogs in the study by Rodrigues Hoffmann, et al. ^24^ were *Streptococcus*, *Diaphrobacter*, and *Sphingomonas*, whereas, *Ralstonia* was the most abundant genera in the nares of healthy dogs. Other studies have identified *Moraxella*, *Cardiobacteriaceae*, *Phyllobacterium*, *Porphyromonas*, *Staphylococcus*, and *Streptococcus* as the predominant genera in the nares of healthy dogs^28,29^. Furthermore, studies have reported a decrease in bacterial diversity and an increase in the relative abundance of the *Staphylococcus* genus on the skin of dogs with dermatitis compared to healthy dogs^24,25,27^. Despite this, *Staphylococcus* is also considered to be the predominant genus on healthy dog’s skin but with varying relative abundance, in addition to *Corynebacterium*, *Kocuria*, *Macrococcus*, *Porphyromonas*, *Propionibacterium*, *Pseudomonas*, and *Streptococcus*^24–27^. Interestingly, a previous study has investigated the effects of MRSA and MSSA colonisation on the lesional skin microbiota of humans with AD, and determined that those colonised with MRSA had reduced microbial diversity compared to MSSA colonisers^30^. This study also identified a decrease in the relative abundance of *Streptococcus*, *Propionibacterium,* and *Corynebacterium* on MRSA-colonised lesional skin^30^. Since various studies have identified a decrease in the diversity and abundance of the skin and nasal microbiota in dogs and humans with MRS and MSS spp. carriage^24,25,30^, it is likely that oral or topical probiotics may be useful in restoring the skin and nasal microbiota of dogs with dermatological conditions^31^. To date, to the best of our knowledge, no studies have investigated whether nasal carriage of MRS and MSS spp. influences the nasal microbiota of dogs with dermatological conditions.

Hence, the aims of this study were to: (a) investigate the nasal carriage of MRS and MSS spp.; (b) determine the risk factors associated with dermatological conditions; (c) explore associations between the resident nasal microbiota and MRS and MSS spp. nasal carriage in dogs with and without dermatological conditions; and (d) identify predictive signalment data and canine nasal microbial genera in dogs with dermatological conditions, in an animal shelter in Brisbane, Queensland, Australia.

## Results

### Study population

Nares of 70 shelter dogs were sampled (n = 186 samples). Medical histories were not available for two dogs, and signalment data was not available for one dog. Twenty-four percent (16/68) of dogs had dermatological conditions over the sampling period. Fifteen of these dogs were diagnosed with dermatological conditions on admission, and one developed a condition during their stay. Eleven of these 16 dogs had skin conditions only, two had ear conditions only, and three had both skin and ear conditions. Seventy-six percent (52/68) of dogs had no dermatological conditions, yet some had other health issues including lameness, vomiting, diarrhoea, renal disease, or kennel cough.

A total of 183 nasal samples from the dogs with available medical histories were taken. Of the baseline nasal samples (n = 68), most dogs (69.1%; 47/68) were sampled within the first 24 h of arrival. A further 8.8% (6/68) were sampled on day two, 14.7% (10/68) were sampled between days three to seven from arrival, while 4.4% (3/68) were sampled between days eight to 14, and 3% (2/68) were sampled at days 18 and 21 (baseline samples after being in the shelter for > 24 h). Although, 23 of the 68 baseline samples corresponded to a unique dog that only stayed in the shelter for 1 day (one baseline sample taken per dog). There were 115 follow-up samples from 45 dogs, where 28 dogs stayed four or more days totalling one baseline and two or more follow-up samples taken per dog, and 17 dogs stayed for two days totalling one baseline and one follow-up sample only per dog.

A higher proportion of nasal samples were taken from dogs with no dermatological conditions (78.7%; 144/183 samples). For dogs with dermatological conditions, 51.3% (20/39) of nasal samples were from dogs with skin conditions only, 30.8% (12/39) with ear conditions only, and 17.9% (7/39) with both skin and ear conditions. Forty-four percent (7/16) of dogs with dermatological conditions were treated with topical antimicrobials only (n = 23 nasal samples). Two percent (1/52) of dogs without dermatological conditions were treated using topical antimicrobials (n = one sample), 13.5% (5/52) were treated using oral antimicrobials (n = 12 samples), and 1% (1/52) were treated using parenteral antimicrobials as a subcutaneous injection (n = one sample). Refer to Tables S10 and S11 of the Supplementary Results for the list of topical and systemic antimicrobials used.

### Methicillin-resistant and methicillin-sensitive* Staphylococcus* spp. nasal carriage

At admission (baseline), 73% (11/15) of dogs with dermatological conditions carried *Staphylococcus* spp. and upon discharge, 100% (16/16) carried at least one *Staphylococcus* spp. Sixty percent (6/10) of these dogs that had two or more samples carried multiple *Staphylococcus* spp. in their nares. Seventy-three percent (38/52) of dogs without dermatological conditions carried *Staphylococcus* spp. and upon discharge, 86.5% (45/52) carried at least one *Staphylococcus* spp. Forty-three percent (15/35) of these dogs that had two or more samples carried multiple *Staphylococcus* spp. in their nares. Overall, irrespective of dermatological conditions, the shelter dogs that stayed two or more days had a higher rate of staphylococci nasal carriage (75.5%; 34/45) upon discharge.

Methicillin-resistant *Staphylococcus* spp. was present in the nares of 26.7% (4/15) of the baseline samples from dogs with dermatological conditions at admission, and throughout the sampling period, 20.5% (8/39) of nasal samples were MRS spp. positive. Of the dogs without dermatological conditions, 11.5% (6/52) had MRS spp. in their nares at admission, with 13.2% (19/144) of nasal samples being positive for MRS spp. over the sampling period. Detailed bacterial culture and antimicrobial susceptibility testing results for all 186 nasal samples are presented in Tables S1, S2, and S3 of the Supplementary Results.

### Risk factors associated with dogs having dermatological conditions

Our univariable models indicated that the variables that should be considered (i.e. *p* ≤ 0.20) in the full multivariable model included sex, dog population, the original location of the dogs before entering any shelter, the dog’s previous shelter location (if any), length of stay at the sampling shelter, the number of days the dogs were in the shelter prior to the baseline swab being taken, antimicrobial usage, nasal carriage and sampling location within the shelter (Table S4 of the Supplementary Results).

Our final multivariable model showed that dogs with dermatological conditions had higher odds of being female [Odds Ratio (OR): 3.73 (95% CI: 2.16–6.44); *p* ≤ 0.001], being present in the shelter two to seven days prior to the baseline swab being taken [OR: 5.02 (95% CI: 1.71–14.75); *p* = 0.003] (Table 1), being positive for MRSA carriage [OR: 11.54 (95% CI: 2.51–53.07); *p* = 0.002], and being treated with antimicrobials [OR: 8.92 (95% CI: 2.18–36.55); *p* = 0.002] compared to dogs without these conditions. Our multivariable results indicated that dogs with dermatological conditions had lower odds of being from the owner surrendered dog population compared to dogs without dermatological conditions [OR: 0.19 (95% CI: 0.05–0.74); *p* = 0.016].

**Table 1:** Multivariable analysis of the risk factors associated with shelter dogs with dermatological conditions.

Variable	Multivariable analysis			Variable	Multivariable analysis		
	n	Odds ratio (95% CI)	*p*-value		n	Odds ratio (95% CI)	*p*-value
Sex				Length of stay (days)			
Male	69	Reference		0–7	67	Reference	
Female	114	3.73 (2.16–6.44)	≤ 0.001	8–14	23	0.55 (0.22–1.36)	0.198
Dog population				15–21	45	0.23 (0.04–1.32)	0.101
Stray dogs	90	Reference		≥ 22	48	1.25 (0.21–7.33)	0.804
Owner surrendered	36	0.19 (0.05–0.74)	0.016	Days in shelter prior to baseline swab being taken			
Humane officer seized	57	2.82 (0.67–11.88)	0.159	≤ 1	119	Reference	
Original location of dogs				2–7	52	5.02 (1.71–14.75)	0.003
Brisbane	33	Reference		> 7	12	2.69 (0.39–18.35)	0.311
North of Brisbane	41	0.76 (0.07–8.31)	0.825	Previous shelter location			
South of Brisbane	25	1.22 (0.24–6.17)	0.239	Shelter one (sampling shelter)	120	Reference	
West of Brisbane	84	0.46 (0.04–4.83)	0.519	Shelter two	28	0.32 (0.01–7.49)	0.482
				Shelter three	35	0.84 (0.23–3.03)	0.793
Antimicrobial usage				Nasal carriage			
No	146	Reference		Culture negative for all bacteria	16	Reference	
Yes	37	8.92 (2.18–36.55)	0.002	Staphylococci culture negative	26	1.00 (0.07–12.99)	0.997
Sampling location within the shelter^a^				MRSP	10	2.70 (0.17–42.72)	0.480
Veterinary clinic	89	Reference		MSSP	74	1.95 (0.70–5.45)	0.201
Veterinary clinic dog holding	19	1.09 (0.35–3.43)	0.877	MRSA	5	11.54 (2.51–53.07)	0.002
Shelters’ dog holdings	63	0.74 (0.44–1.26)	0.272	MSSA	16	0.19 (0.02–1.90)	0.158
Shelter’s adoption centre^b^	12	NA		MR-CoNS	12	1.77 (0.11–12.98)	0.894
				MS-CoNS	24	0.81 (0.11–5.86)	0.835

For all variables in the multivariable model, there were 183 nasal samples included.

*n* the number of individual samples per variable.

*MRSP* methicillin-resistant *S*. *pseudintermedius*, *MSSP* methicillin-sensitive *S*. *pseudintermedius*, *MRSA* methicillin-resistant *S*. *aureus*, *MSSA* methicillin-sensitive *S*. *aureus*, *MR-CoNS* methicillin-resistant coagulase-negative staphylococci, *MS-CoNS* methicillin-sensitive coagulase-negative staphylococci.

^a^The shelter includes a veterinary clinic, a veterinary clinic dog holding for dogs still requiring veterinary care, dog holdings, and an adoption centre.

^b^The shelters’ adoption centre was omitted from the multivariable model as it predicts failure perfectly (no dogs with dermatological conditions located at the adoption centre). *N* the number of total samples per variable, *n* the number of individual samples per variable, *NA* not applicable.

### Profiling nasal microbiota diversity and abundance

#### Nasal microbiota study population

Of the 52 samples, 28 dogs had one baseline nasal sample only, seven dogs had one baseline and one follow-up sample each, one dog had one baseline and two follow-up samples, one dog had one follow-up sample, and three dogs had only two follow-up samples each (no baseline samples). Thirteen of these 52 samples were from ten dogs with dermatological conditions (n = six baseline and seven follow-up nasal samples). Thirty-nine of these 52 samples were from 30 dogs without those conditions (n = 28 baseline and 11 follow-up samples). Four dogs with dermatological conditions (n = one baseline and five follow-up samples) corresponded with a topical antimicrobial treatment. Two dogs without dermatological conditions were treated with oral antimicrobials (n = two baseline and one follow-up sample).

A total of 3,613,386 sequences were amplified from all 52 nasal samples, with 86.7% (3,132,642/3,613,386 sequences) passing quality control. The minimum reads per sample were 9,461, while the maximum reads per sample were 206,747. These sequence reads were then taxonomically classified across 895 amplicon sequence variants (ASVs), with 562 being identified at the genus level. At the genus level there were 82 uncultured genera and 18 unknown/unassigned genera, respectively. The nasal carriage status of dogs with and without dermatological conditions are presented in Table S5 of the Supplementary Results.

#### Nasal microbiota of dogs with and without dermatological conditions

##### Relative abundance, alpha diversity, and core microbiota

**Figure 1:**
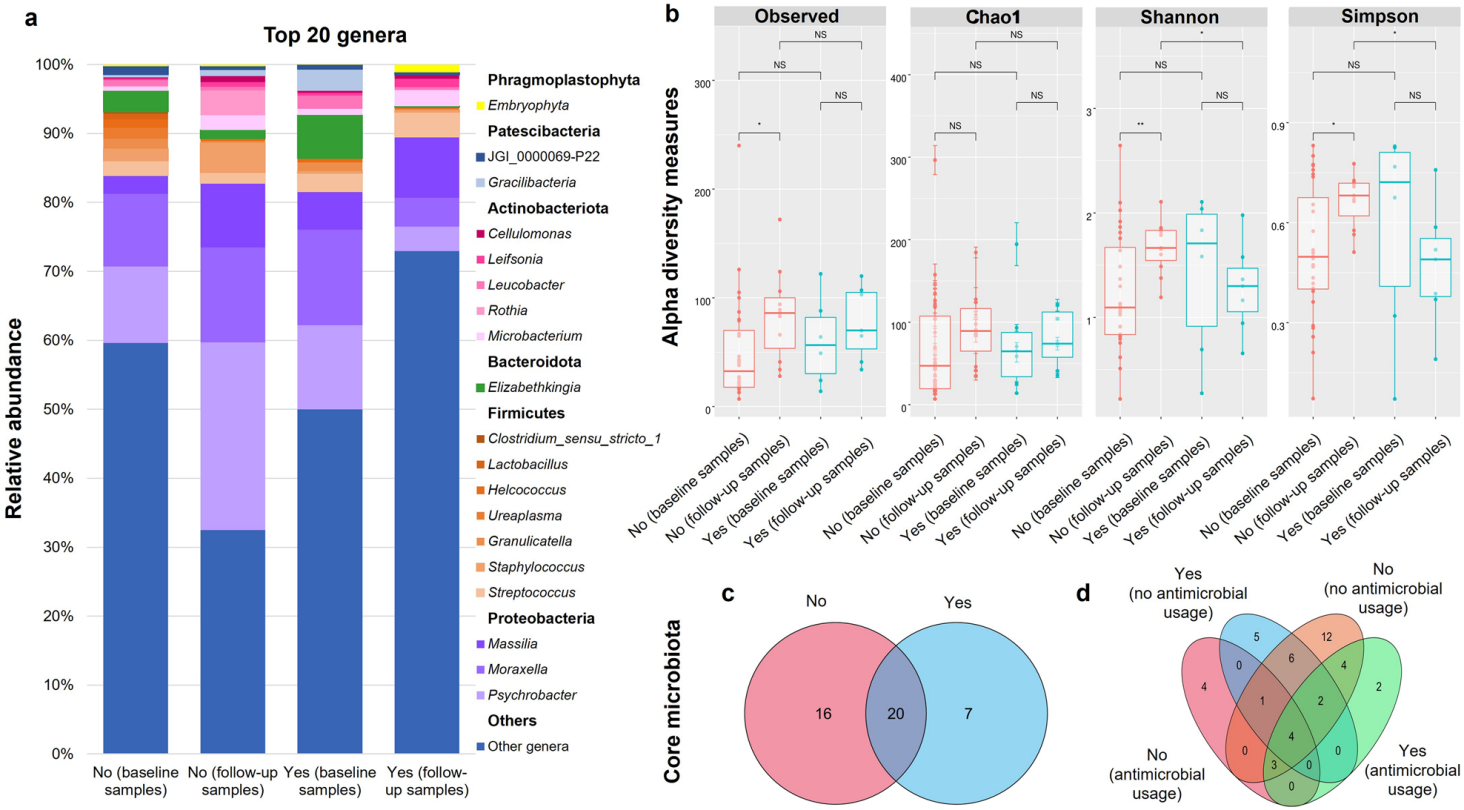
Microbiota analysis of nasal samples from dogs with (yes) and without (no) dermatological conditions displaying **(a)** relative abundance (%) of the top 20 genera for the baseline and follow-up samples; **(b)** alpha diversity plots of observed richness, Chao1, Shannon Index, and Simpson’s Index for the baseline and follow-up samples using Wilcoxon rank-sum test significance [p > 0.05 = NS (not significant), *p < 0.05, **p < 0.01]; **(c)** core microbiota at genus level from nasal samples of dogs with and without dermatological conditions; **(d)** core microbiota at genus level of nasal samples from dogs with dermatological conditions that were prescribed antimicrobials (yes—antimicrobial usage) and not prescribed antimicrobials (yes—no antimicrobial usage) and from dogs without those conditions prescribed antimicrobials (no—antimicrobial usage) and not prescribed antimicrobials (no—no antimicrobial usage).

In all nasal microbiota samples (n = 52), *Psychrobacter*, *Moraxella*, *Massilia*, *Elizabethkingia,* and *Streptococcus* were the top five genera, followed by the genus *Staphylococcus* (Fig. 1a). In the follow-up samples of dogs without dermatological conditions, *Psychrobacter*, *Moraxella*, *Massilia*, and *Staphylococcus* were the dominant genera (Fig. 1a). *Staphylococcus* relative abundance was low for dogs with dermatological conditions for both sample timings, while *Streptococcus* relative abundance was higher (Fig. 1a).

There were no significant differences (*p* > 0.05) in the alpha diversity for dogs with dermatological conditions between their baseline and follow-up samples (Fig. 1b). For dogs without dermatological conditions, the observed richness of the follow-up nasal samples was statistically higher (*p* < 0.05), compared to the baseline samples (Fig. 1b). Also, between the baseline and follow-up samples, Shannon’s and Simpson’s diversity indices differed significantly (*p* < 0.01 and *p* < 0.05, respectively) (Fig. 1b). Additionally, when comparing the follow-up samples of dogs with and without dermatological conditions, the differences in both Shannon’s and Simpson’s diversity indices were statistically significant (*p* < 0.05) (Fig. 1b). There was also no significant difference observed in alpha diversities between dog groups for nasal carriage (MRS spp., MSS spp., and culture negative for MRS and MSS spp.), and antimicrobial usage (Figs. S1 and S2 of the Supplementary Results).

When focusing on sequence reads with ≥ 1% relative abundance, only 44 genera were identified and used for core microbiota analyses to determine shared and unique genera, irrespective of the sample timing. When comparing the canine nasal microbial genera in dogs with and without dermatological conditions, seven genera including *Acidibacter*, *Bacteroides*, *Chloroplast*, *Cladosporium*, *Faecalibacterium*, *Serratia*, and an unclassified Solirubrobacterales referred to as 67–14 were unique to the nares of dogs with dermatological conditions (Fig. 1c). Sixteen genera were unique to dogs without dermatological conditions, while 20 genera were shared (core genera) between the dog groups (Fig. 1c). *Chloroplast* and *Cladosporium* were uniquely identified in the nares of dogs with dermatological conditions treated with antimicrobials (Fig. 1d), whilst *Acidibacter*, *Bacteroides*, *Faecalibacterium*, *Serratia,* and an unclassified Solirubrobacterales were unique to dogs with dermatological conditions not treated with antimicrobials (Fig. 1d). Additionally, an UpSet plot was used to determine the core microbiota present for nasal carriage of dogs with and without dermatological conditions (Fig. S3 of the Supplementary Results). The full list of shared and unique genera for dogs with and without dermatological conditions for the core microbiota analyses are presented in Tables S6, S7, and S8 of the Supplementary Results.

##### Beta diversity

**Figure 2:**
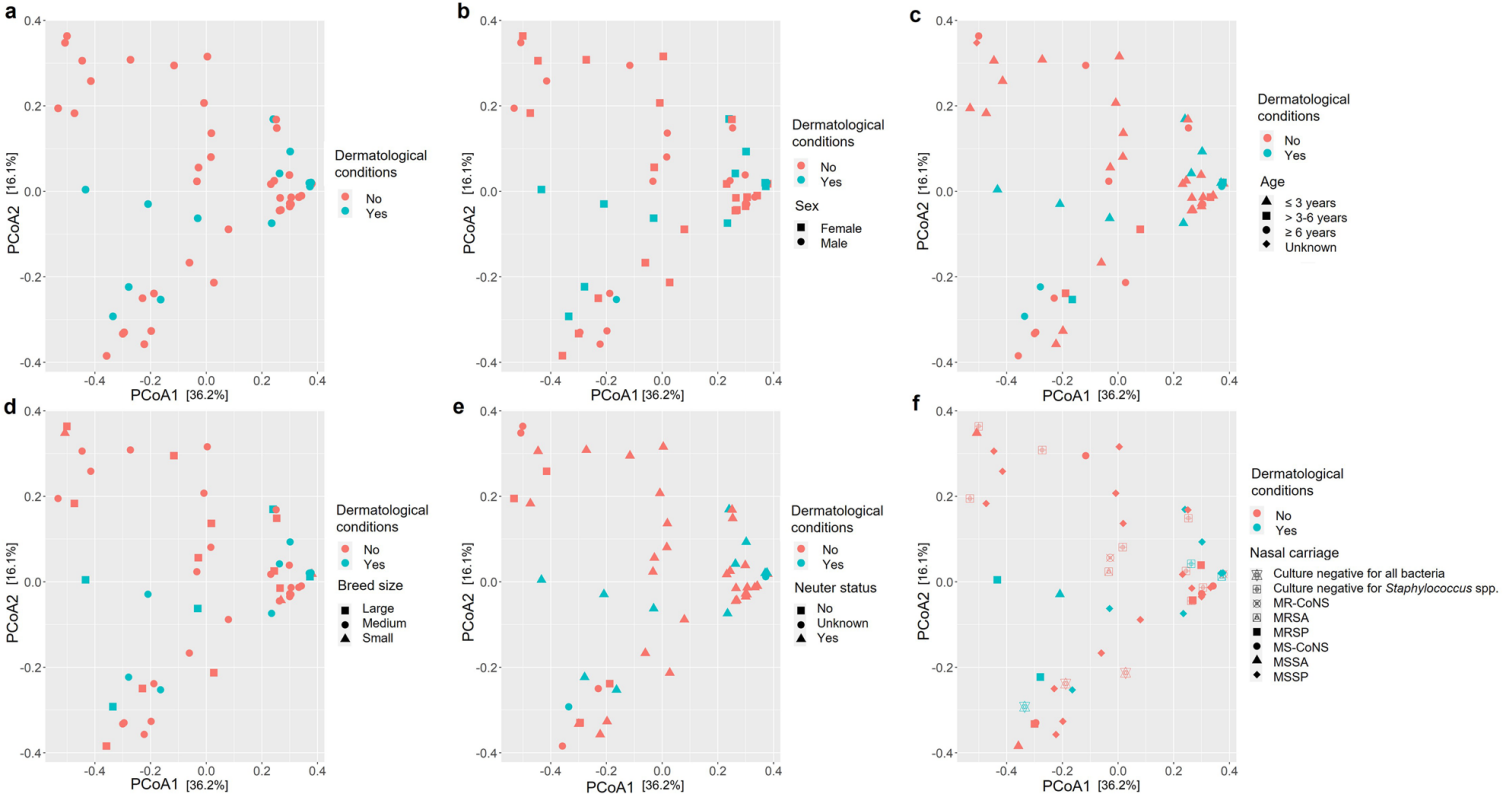
PCoA plots with Bray–Curtis displaying the amplicon sequence variants (ASVs) from the nasal samples (n = 52) from **(a)** dogs with (yes) and without (no) dermatological conditions and comparing that to **(b)** sex; **(c)** age; **(d)** breed size; **(e)** neuter status; and **(f)** nasal carriage represented by the corresponding symbols per plot.

Principal coordinate analysis (PCoA) based on Bray–Curtis dissimilarity metrics depicted 52.3% of the microbial communities between the nasal samples from dogs with and without dermatological conditions, and the signalment variables (Fig. 2). No clustering was observed based on condition alone, in addition to no clustering for sex, age, breed size, neuter status and nasal carriage (Fig. 2a–f), and antimicrobial usage (Fig. S2b of the Supplementary Results). However, without accounting for the condition, the microbiota of dogs which cultured negative for all bacteria in the nares on the selective agar plates were less dissimilar, clustering together in the lowest horizontal quadrant of the PCoA plot (Fig. 2f).

### Associations between canine dermatological conditions, signalment, and nasal microbiota diversity

From a total of 580 variables, 30 of these were selected using the information value results. For Model 1, the 34 baseline nasal microbiota samples included 17.6% (6/34) of samples from dogs with dermatological conditions and 82.4% (28/34) of samples from dogs without those conditions. For Model 2, the 18 follow-up nasal microbiota samples included 38.9% (7/18) of samples from dogs with dermatological conditions and 61.1% (11/18) of samples from dogs without those conditions. Both models were highly accurate in being able to classify variables as predictors of dermatological conditions in shelter dogs (Model 1 and Model 2: AUC = 1; sensitivity and specificity = 1). The coefficient values for both models are presented in Table S9, in Supplementary Results.

**Figure 3:**
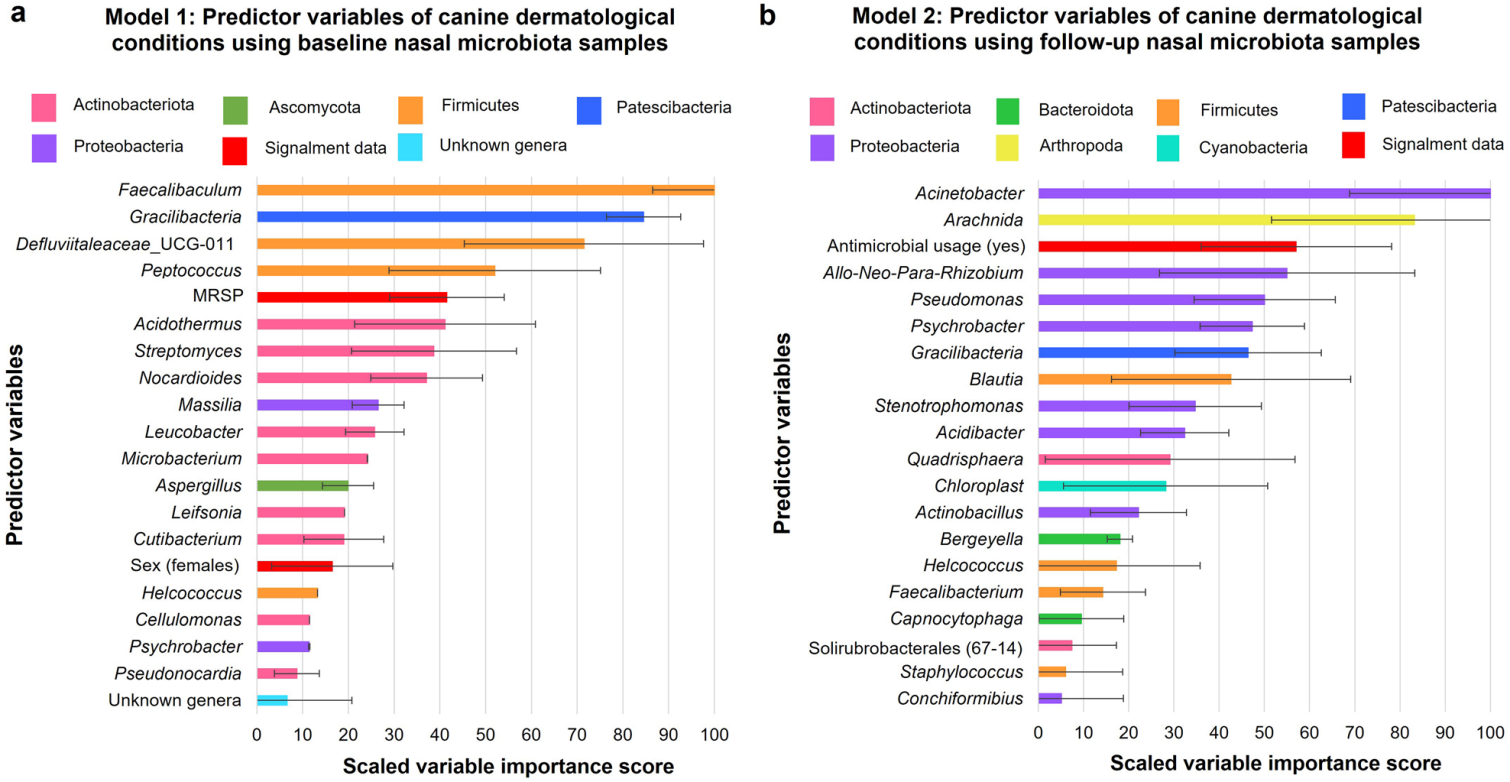
Barplots representing the top 20 most predictive variables identified by the elastic net logistic regression model for dogs with dermatological conditions. **(a)** Model 1 used only the baseline nasal samples and signalment data; and **(b)** Model 2 used only the follow-up nasal samples and signalment data. The whiskers (error bars) represent the standard deviations from training the selected features (variables) from the highest AUC models which were repeated 10 times (AUC ≥ 0.7). The coloured bars represent the different phyla. Allorhizobuim–Neorhizobium–Pararhizobium–Rhizobium was abbreviated to Allo-Neo-Para-Rhizobium in this graph. *MRSP* methicillin-resistant *S. pseudintermedius*.

In Model 1, *Faecalibaculum*, *Gracilibacteria*, and *Defluviitaleaceaea*_UCD-011 were identified as the most predictive genera in the nasal microbiota (Fig. 3a). Sex (females) and MRSP nasal carriage were the only signalment variables included for Model 1 (Fig. 3a). For Model 2, the variables considered to be most predictive of dermatological conditions in shelter dogs’ nares were *Acinetobacter* and *Arachnida* genera, followed by antimicrobial usage (Fig. 3b). Topical antimicrobial usage corresponded to 16.6% (1/6) of nasal microbiota samples from dogs with dermatological conditions in Model 1. For Model 2, 71.4% (5/7) of nasal microbiota samples from dogs with dermatological conditions corresponded to topical antimicrobial usage (Refer to Tables S10 and S11 of the Supplementary Results for the list of antimicrobials used). Repeating the selected variables from the final models (represented as error bars in Fig. 3a,b), revealed that the variable importance scores differed highly depending on each individual repeated model.

#### Co-occurrence networks based on elastic net logistic regression model outcomes

**Figure 4:**
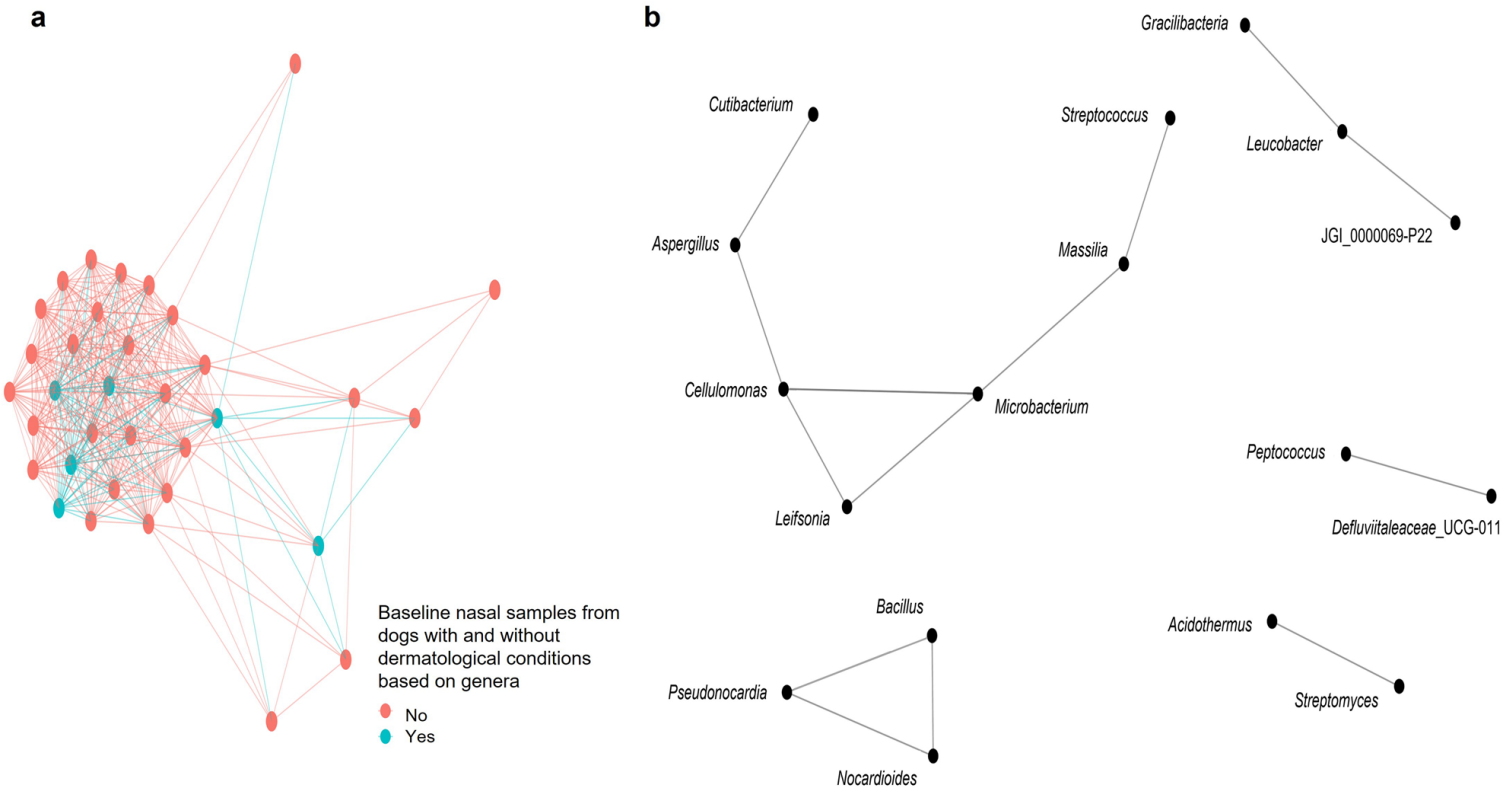
Co-occurrence network analysis using the genera with coefficient values from the elastic net logistic regression Model 1 (baseline nasal microbiota samples) using Bray–Curtis dissimilarly and maximum distance of 0.8 displaying the connections between **(a)** baseline nasal microbiota samples of dogs with (yes) and without (no) dermatological conditions and **(b)** the genera.

**Figure 5:**
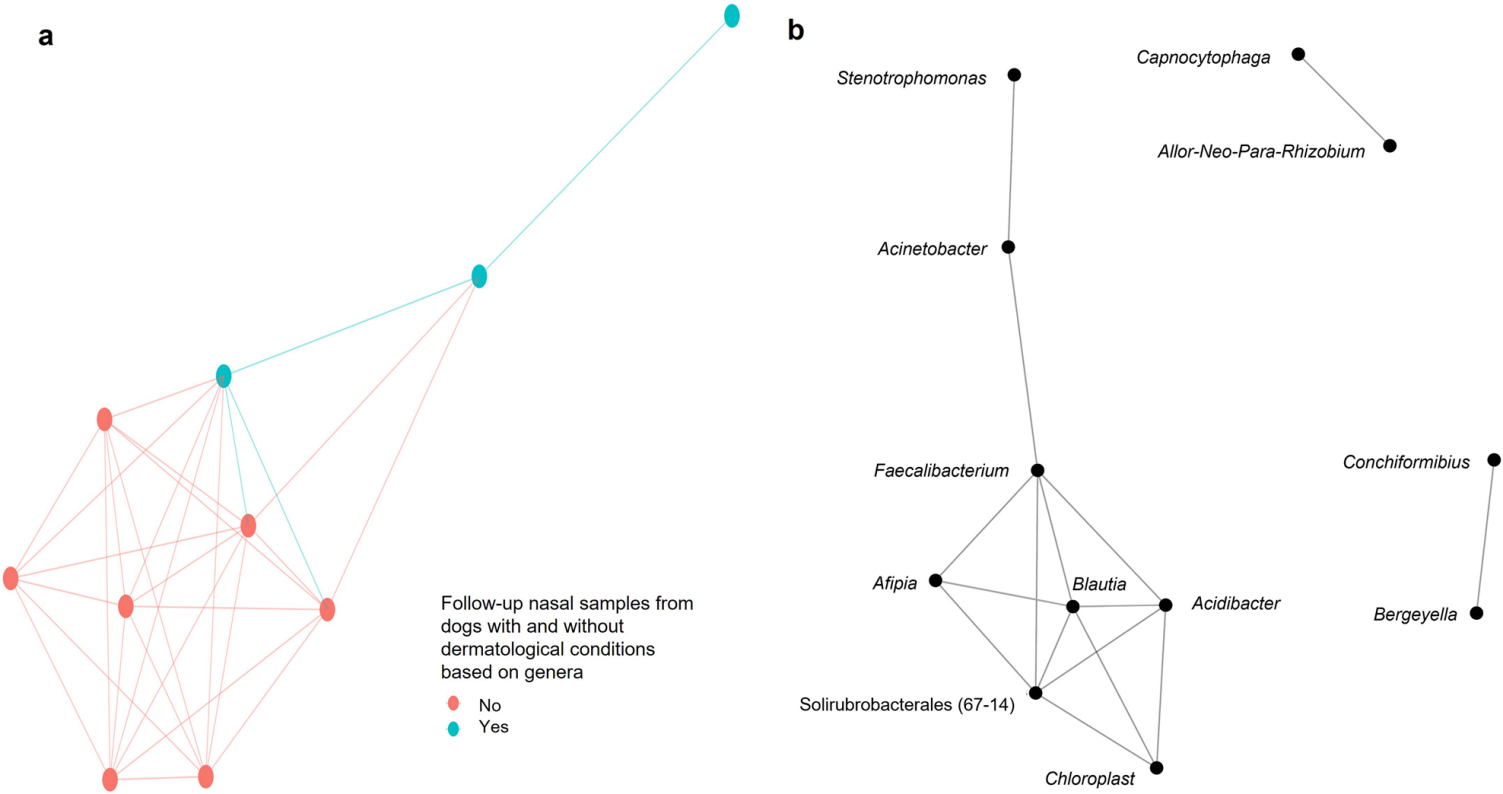
Co-occurrence network analysis using the genera with coefficient values from the elastic net logistic regression Model 2 (follow-up nasal microbiota samples) using Bray–Curtis dissimilarly and maximum distance of 0.8 displaying the connections between **(a)** follow-up nasal microbiota samples of dogs with (yes) and without (no) dermatological conditions and **(b)** the genera. Allorhizobuim–Neorhizobium–Pararhizobium–Rhizobium was abbreviated to Allo-Neo-Para-Rhizobium in this graph.

Using nasal microbiota data collected at baseline (Model 1) and follow-up (Model 2), we were able to identify a total of 25 and 20 genera, respectively. Using those genera, our results indicate that the nasal microbiota at baseline (Model 1) were similar for dogs with and without dermatological conditions (Fig. 4a). At follow-up (Model 2), the nasal microbiota were dissimilar for dogs with and without dermatological conditions (Fig. 5a).

When investigating the co-occurrence of the nasal microbiota at the genera level (not dog group), of the 25 genera identified in Model 1, only 17 remained in the network, with eight of those being positively correlated to dogs with dermatological conditions (Fig. 4b). There were connections between both the positively and negatively associated genera, based on the coefficient output from Model 1 (Fig. 4b). For instance, *Bacillus* (negative coefficient) co-occurred with *Nocardioides,* and *Pseudonocardia* (both positive coefficients), within its own cluster (Fig. 4b). A total of 12 out of 20 genera from Model 2 were retained, with eight correlating to dogs with dermatological conditions (Fig. 5b). For the follow-up samples, all of the positively associated genera from Model 2 formed one cluster, with the four negatively associated genera forming two separate clusters (Fig. 5b).

## Discussion

In this study, we compared the nasal microbiota of shelter dogs carrying MRS and MSS spp., with and without dermatological conditions, using conventional microbiota bioinformatics analyses and machine learning techniques. Significant differences in nasal microbiota diversity and abundance were only observed based on dog group and sample timing using standard microbiota analyses. Utilising elastic net logistic regression, we identified nasal microbial genera of shelter dogs that were predictive of dermatological conditions at baseline and follow-up, whilst accounting for signalment, nasal carriage data, and antimicrobial usage.

Our study shows that upon admission and throughout the study period when accounting for all nasal samples, dogs with dermatological conditions had a higher isolation of MRS spp. in the nares compared to dogs without those conditions. The high isolation of MRS spp. in dogs with dermatological conditions is concerning due to the potential for the carriage of nasal MRS spp. to cause secondary bacterial skin infections. This has been identified in children with AD, whereby, the nasal MRSA-colonising isolates were clonally related to the MRSA-infecting isolates^22^. Additionally, as dogs with dermatological conditions often have secondary bacterial infections^2^, decolonisation of the MRS spp. may be beneficial in reducing the risk of these bacteria, particularly MRSP, in causing skin and ear infections in shelter dogs.

When investigating the staphylococci nasal carriage as risk factors for dogs with dermatological conditions using all samples, only MRSA was significant. This was surprising as *S*. *pseudintermedius* is the most frequently isolated bacterium from dogs with skin diseases^18,32^. However, as there was a large 95% confidence interval, and the result was only represented by two dogs, this finding should be interpreted cautiously. Dogs with dermatological conditions in this study also had higher odds of being female compared to dogs without those conditions. This is contradictory to other studies investigating the risk factors of dogs with AD and otitis externa, which reported either no differences between sexes or that males had a higher risk^33–37^. The difference in target populations between our study (shelter animals) and other studies (veterinary clinics or insurance databases)^33–37^, may partially explain the sex differences. The findings that antimicrobials were identified as a risk factor for dogs with dermatological conditions were expected, in that topical antimicrobials such as shampoos and ointments are often prescribed for dogs with pyoderma and otitis externa^38,39^. The significantly lower odds of dogs with dermatological conditions being from an “owner surrendered dog population” may be due to the care and treatment of dogs before entering the shelter.

The higher relative abundance of *Staphylococcus* spp. in the nares of dogs without dermatological conditions was contradictory to other studies investigating the nasal and skin microbiota of dogs with allergic or AD^24,25,27^. Our results echo those reported by Rodrigues Hoffmann, et al. ^24^, who showed that the nares of dogs with allergic dermatitis were predominantly colonised by *Streptococcus* spp. Additionally, the significant differences in alpha diversities between baseline and follow-up nasal samples from dogs without dermatological conditions suggests that length of stay is likely to influence the diversity and abundance of dog’s nasal microbiota. This was also evident when comparing follow-up samples of dogs with and without dermatological conditions.

Regarding the microbiota analyses of this study, we are the first to our knowledge to report core microbiota analyses while investigating *Staphylococcus* spp. nasal carriage and antimicrobial therapy effect on the nasal microbiota. To date, only two studies have examined the skin microbiota in dogs with AD before and after antimicrobial therapy^25,27^, where core microbiota analyses were not reported. However, our beta diversity plots which identified no clustering between the nasal microbiota samples from dogs with and without dermatological conditions (Fig. 2), were similar to a previously reported finding by Rodrigues Hoffmann, et al. ^24^, who investigated the microbiota of allergic and healthy dogs.

As our alpha diversity measures demonstrated a significant difference between dogs with and without dermatological conditions for the follow-up samples, we adopted an ENR machine learning approach to further explore this association^40–43^. This allowed for the identification of predictive nasal microbiota genera of dermatological conditions accounting for important multicollinear variables^40–43^. The ENR approach was also selected for this investigation due to its ability to effectively analyse data with a small sample size and a large number of variables commonly represented in microbial data^44^. The ENR models identified predictive genera of disease, and along with the co-occurrence networks, demonstrated that there was a relationship between multiple canine nasal microbial genera in dogs with dermatological conditions. Furthermore, nasal carriage at the species level was unable to be investigated for the alpha diversities using conventional microbiota analyses due to the low sample size. However, when using ENR, MRSP was identified as a predictive signalment variable, along with female dogs. This is an important finding as *S*. *pseudintermedius* causes both pyoderma and otitis externa, and MRSP is becoming more common in pyoderma cases^45–47^.

Additionally, this machine learning analyses shows that there were changes in predictive signalment data and microbial genera in the nasal microbiota between baseline (Model 1) and follow-up (Model 2) samples, in that we identified differences in signalment and predictive genera and genera ranking in the two ENR models. The co-occurrence networks indicated that the nasal microbiota of the dog groups was more similar for the baseline samples. This is despite the dogs originally being from different geographical locations and having different medical histories upon arrival. The changes in microbial genera identified in the ENR models were also indicated in the co-occurrence network for the follow-up samples suggesting that factors within the shelter altered the microbiota between the dog groups. The co-occurrence networks further demonstrated the dissimilarity of the nasal microbial genera, as the connections between the genera were based on whether the genera positively or negatively predicted dermatological conditions for the follow-up samples. Due to this dissimilarity and connections between the genera, our findings for the follow-up samples suggest antimicrobial usage is the only non-microbial predictor in ENR Model 2, highlighting the significance of antimicrobial usage in dogs with dermatological conditions as a factor influencing the nasal microbiota in shelter dogs. Overall, combining signalment, MRS and MSS spp. nasal carriage, and antimicrobial treatment with nasal microbial data in machine learning aids in understanding changes in the importance of those variables in dogs with dermatological conditions.

It should be noted that our findings may be influenced by the fact that only 28 dogs were sampled for ≥ 4 days, a larger longitudinal study was not possible due to dogs being moved through the shelter or being transferred to another shelter faster than expected. Additionally, some dogs were also unable to be resampled after the baseline sample was taken due to behavioural and/or medical concerns, thus limiting the opportunities for more follow-up samples to be collected. Furthermore, due to one swab being taken per dog from both nostrils, only 52 nasal samples were submitted for 16S rRNA gene amplicon sequencing, due to DNA quantities. Only one sample had less than the minimum sequence reads per sample threshold of 10,000 (9,461 reads), and thus it is unlikely that the bioinformatics analyses would have been biased. Future studies should aim to collect two nasal swabs per dog each sampling time which could be pooled to increase DNA yield^48^, however, this was beyond the scope of our ethics agreement. Also, as this study was conducted at an animal shelter undertaking their normal routines, for ethical reasons it was not feasible to control for antimicrobial usage in the sampled dogs. Yet after accounting for antimicrobial usage, ENR Model 2 demonstrated the importance of this variable for the nasal microbiota of dogs with dermatological conditions and changes in predictive genera. Thus, when interpreting these results, it is important to identify the potential effect of antimicrobials on the nasal microbiota, in addition to canine dermatological conditions. Lastly, 69.1% (47/68) of dogs were sampled within the first 24 h of arrival at the shelter, while this was not the case for 30.9% (21/68) of dogs. This was due to the busyness of the shelter. Future studies could further investigate the relationship between MRS and MSS spp. nasal and skin carriage in dogs with allergic dermatitis or AD whilst accounting for disease severity in both animal shelters and veterinary clinics and shelter/practice-based data. Indeed, two human-based studies have observed that persistent nasal *S*. *aureus* carriers experienced more severe AD^49,50^. Additionally, future studies could use machine learning techniques like ENR models to investigate predictive genera of dogs with skin and ear conditions from their corresponding microbiotas, to determine if there are any differences between body sites or condition types. This analysis also has the potential to incorporate results from gene marker sequencing or whole-genome sequencing of cultured MRS and MSS spp. isolates to better understand the relationship between the microbiota and the organisms’ genetics.

By using ENR, significant associations between MRSP nasal carriage and dogs with dermatological conditions were revealed, whilst accounting for genera in the nares at baseline. This was despite our overall results showing no association between MRS and MSS spp. nasal carriage and microbiota abundance and diversity, using standard microbiota bioinformatics analyses. Additionally, due to the continual isolation of MRS spp. throughout the dog’s time at the shelter, our study highlights the importance of determining if decolonisation therapies are necessary to reduce the infection risk of dogs with dermatological conditions. The ENR models not only identified similar signalment risk factors indicating their importance for dermatological conditions in shelter dogs such as antimicrobial usage but also highlighted changes in predictive genera between the baseline and follow-up nasal microbiota samples. Lastly, as our study showed that the follow-up nasal microbiota samples were statistically different between dog groups, indicating lowered diversity and abundance for dogs with dermatological conditions, these dogs may benefit from the use of probiotic treatments to restore the nasal microbiota. The clinical relevance of such an approach deserves further investigation.

## Methods

### Ethics statement

This animal study was approved by the Production and Companion Animals ethics committee, School of Veterinary Science, The University of Queensland (The University of Queensland Animal Ethics SVS/487/15/KIBBLE) and was performed in accordance with all relevant guidelines and regulations. The methods described in the current study and reported results were compliant with ARRIVE guidelines. Consent was given by the animal shelter to sample the animals housed within the shelter.

### Animal sample collection

Nasal swabs were taken from August 13th to November 15th, 2019, using convenience sampling of the shelter dogs for the purpose of identifying MRS and MSS spp. carriage and characterising the resident microbiota. A baseline sample was taken ideally within 24 h of arrival at the animal shelter at either a veterinary check-up or desexing. However, if this did not happen, the length of time since admission was recorded and the animal was still included in the study. Follow-up samples were taken twice a week until discharge (e.g., adoption, foster, moved to another animal shelter, or humane euthanasia).

Both nares of the dogs were swabbed using one ‘Eswab’ swab (481CE; Copan Diagnostics Inc., California, USA), per sampling timepoint by inserting the swab into the nostril and rotating carefully. Gloves were changed between dogs to prevent cross-contamination during sampling. Thereafter, swabs were aseptically snapped into liquid Amies medium (1 mL) (Copan Diagnostics Inc., California, USA). The baseline samples were stored (4 °C; for ≤ 48 h) at the shelter and collected on the same day as all follow-up samples. Samples were transported (4 °C) to the laboratory for processing. Bacterial isolation was conducted within the 48 h of collection, whereas, identification of nasal MRS and MSS spp., antimicrobial susceptibility testing, and identification of the *mecA* gene which confers methicillin resistance in *Staphylococcus* spp. were conducted in batches (refer to Supplementary Methods).

### Data collection

Medical history and signalment data were retrospectively extracted from the animal shelter’s database for the sampling period. Dogs were grouped into whether they had or were free of a dermatological condition. For dogs with veterinary medical records that included terms such as otitis externa (bacterial and yeast), pruritus, interdigital dermatitis, chronic dermatitis, flea allergic dermatitis, erythema, lichenification, hyperpigmentation, excoriation, alopecia, papules, and sarcoptic mange mite, were identified as having a dermatological condition. Information regarding antimicrobial usage was collected and identified by manually reviewing all veterinary consultation notes. Veterinary notes were correlated to the date that the swabs were taken. Refer to Supplementary Methods for the full list of search terms.

For each dog, the signalment data included: estimations of birth date or age unless available using microchip data, sex, breed, neuter status, the date the dogs entered and left the animal shelter, and the dog population (stray, owner surrendered, and humane officer seized/surrendered). The location of where the dog originated from and whether the dog was located at a different animal shelter prior to being sampled were also included.

### DNA extraction for microbiota analysis

Nasal samples were processed for microbiota analyses by thawing at 4 °C and centrifuging (13,500×*g*; 4 °C; 5 min) to pellet the cells. Pellets were washed with 1 × phosphate buffered saline (PBS; 1 mL) and stored (−20 °C) until the DNA was extracted as described by Yao, et al. ^51^ using 600 μL lysis buffer (50 mM Tris–HCl at pH 8.0, 4% sodium dodecyl sulphate, 500 mM NaCl, 50 mM EDTA).

Total genomic DNA (gDNA) was extracted using the Maxwell®16 Instrument (Promega, Wisconsin, USA) and the Maxwell^®^ 16 SEV Cell DNA Purification Kit (AS1020, Promega, Wisconsin, USA), as per the manufacturer’s instructions. The DNA quality and concentration was determined using the NanoDrop™ 8000 Spectrophotometer (Thermo Fisher Scientific, Massachusetts, USA), and samples with low DNA concentrations underwent ethanol precipitation. Only samples with a DNA concentration of ≥ 1.88 µg/µL were submitted for 16S rRNA gene amplicon sequencing.

### Microbiota sequencing and bioinformatics analysis

16S rRNA gene amplicon sequencing of 52 of the 183 nasal samples were selected based on DNA quantity and was carried out by the Australian Centre for Ecogenomics (ACE; the University of Queensland, Queensland, Australia), using the Illumina MiSeq Platform (Illumina, California, USA), where the V6 to V8 variable region was targeted using the primers 926F and 1392wR^52^. The generated sequence reads were imported into QIIME 2^53^ and denoised with DADA2^54^. The SILVA rRNA database^55^ was used for the taxonomic classification of representative sequences.

In R statistical software v4.1.2, the data was rarefied to an analysable 9,445 reads per sample and alpha diversity parameters (observed richness, Shannon’s Index, Chao1, and Simpson’s Index) were performed with respect to the dog groups’ nasal samples and sample timing, nasal carriage, and antimicrobial usage. Wilcoxon rank-sum test was carried out for each alpha diversity parameter to determine whether the results were significant. Principal coordinates analysis (PCoA; beta diversity) was conducted to determine the microbial diversity between the dog groups’ nasal samples regardless of sample timing. The relative abundances of the top 20 genera were identified and then visualised as a bar chart using Excel^56^ with respect to the dog groups’ nasal samples and sample timing. Using Venn diagrams^57^ and an UpSet plot^58^, the core microbiota at the genus level were displayed for the dog groups, including only genera with a relative abundance of ≥ 1%. For the full list of R packages, refer to Supplementary Methods.

### Statistical analyses

#### Risk factor analysis

Using Stata v17.0 (Stata Corporation, Texas, USA), a Bernoulli logistic regression model was used to identify risk factors associated with dogs with dermatological conditions (N = 183; the outcome of interest) and the variables of interest at the shelter whilst adjusting for the resampling of the dogs. For the univariable model, a cut-off overall *p-*value of ≤ 0.20 per variable was considered significant and were retained in the multivariable model. To identify confounders, a manual backward stepwise variable selection procedure was conducted. If a removed variable had a ≥ 25% change on any other variables’ coefficient, then that variable was retained in the model as a confounder. A *p*-value of 0.05 in the multivariable analysis was considered significant. The smallest estimate of the Akaike information criterion (AIC) was used to determine the final multivariable model.

##### Predictive modelling

Elastic net logistic regression (ENR) models were conducted to investigate the associations between signalment, staphylococci nasal carriage, antimicrobial treatment, and the relative abundances of the nasal microbiota identified at the genus level (n = 580 predictor variables) in shelters dogs with dermatological conditions (n = 52 nasal samples; the outcome of interest). Two models were run using only the baseline nasal samples (n = 34 nasal samples; Model 1) and only the follow-up nasal samples (n = 18 nasal samples; Model 2). For both ENR models, the variables included sex, neuter status, breed size, age, nasal carriage, and antimicrobial usage as dummy variables (if required), along with the relative abundances of 562 genera, totalling 580 variables. A 70/30 training/testing dataset split was used for both models. The training dataset’s predictor variables were evaluated using information value^59^ to select variables to include in a reduced training and testing model. The selected variables were then trained with a repeated ten-fold cross validation (CV) with five repeats and underwent pre-processing to further normalise the data using “nzv”, “centre” and “scale”^60^. The accuracy of the model was determined using the reduced testing dataset using the area under the (receiver operation characteristics) curve (AUC) for Model 1 and Model 2, separately. Models with an AUC of ≥ 0.7 were classified as acceptable models^61^. To identify the top 20 predictors of canine dermatological conditions, the scaled variable importance scores which ranked the absolute values of the coefficients of the selected variables from the training model were visualised using barplots colour-coded at phylum level. The final Model 1 and Model 2 selected variables were repeated 10 times by shuffling the dataset each time to calculate the standard deviation from the variable importance scores, represented as error bars in the barplots. Only the repeated models with an AUC ≥ 0.7 were included. The analysis was performed using the caret^62^, information^63^, information value^59^, metrics^64^, and glmnet^65^ packages in R statistical software v4.1.2.

##### Co-occurrence network analysis based on the predictive modelling outcomes

To better understand the associations of genera with coefficient values identified in the final ENR Model 1 and Model 2 from the nasal samples of dogs with and without dermatological conditions, co-occurrence networks were created. Two networks were created per model using Bray–Curtis dissimilarity and a maximum distance of 0.8. The first network displayed the nasal microbiota samples associated with dogs with or without dermatological conditions, connected based on the genera from Model 1 and Model 2, separately. The second network displayed the connections between the genera using samples from dogs with and without dermatological conditions from Model 1 and Model 2. All networks were created in R statistical software v4.1.2 using igraph version 1.3.2^66^ and phyloseq^67^.

## Supplementary Information

Supplementary Information.

## Data Availability

The sequences generated during and/or analysed during the current study are available in the European Nucleotide Archive of the European Bioinformatics Institute (EBI), under the accession number ‘PRJEB58552’ (https://www.ebi.ac.uk/ena/browser/view/PRJEB58552). All other datasets generated during and/or analysed during the current study are not publicly available due to the data sharing consent from the animal shelter but are available from the corresponding authors on reasonable request.

## References

[1] Newbury S, Moriello KA (2006). Skin diseases of animals in shelters: Triage strategy and treatment recommendations for common diseases. Vet. Clin. N. Am. Small Anim..

[2] DeBoer DJ, Marsella R (2001). The ACVD task force on canine atopic dermatitis (XII): The relationship of cutaneous infections to the pathogenesis and clinical course of canine atopic dermatitis. Vet. Immunol. Immunopathol..

[3] Hillier A (2014). Guidelines for the diagnosis and antimicrobial therapy of canine superficial bacterial folliculitis (Antimicrobial Guidelines Working Group of the International Society for Companion Animal Infectious Diseases). Vet. Dermatol..

[4] János D (2021). Carriage of multidrug resistance staphylococci in shelter dogs in Timisoara, Romania. Antibiotics.

[5] Zur G, Gurevich B, Elad D (2016). Prior antimicrobial use as a risk factor for resistance in selected *Staphylococcus **pseudintermedius* isolates from the skin and ears of dogs. Vet. Dermatol..

[6] Kjellman EE, Slettemeås JS, Small H, Sunde M (2015). Methicillin-resistant *Staphylococcus **pseudintermedius* (MRSP) from healthy dogs in Norway—Occurrence, genotypes and comparison to clinical MRSP. MicrobiologyOpen.

[7] Boost MV, O'Donoghue MM, James A (2008). Prevalence of *Staphylococcus aureus* carriage among dogs and their owners. Epidemiol. Infect..

[8] Gingrich EN, Kurt T, Hyatt DR, Lappin MR, Ruch-Gallie R (2011). Prevalence of methicillin-resistant staphylococci in northern Colorado shelter animals. J. Vet. Diagn. Invest..

[9] Machado AB, Machado MFR, Picoli SU (2017). An investigation of methicillin-resistant *Staphylococcus **pseudintermedius* (MRSP) in domestic and shelter dogs in Montenegro (RS-Brazil). Rev. Bras. Saude Prod. Anim..

[10] Huang TM, Chou CC (2019). Methicillin-sensitive and methicillin-resistant *Staphylococcus aureus* strains and their toxin genes in the nostrils of dogs and workers at an animal shelter. J. Appl. Microbiol..

[11] Huerta B (2011). Risk factors associated with the antimicrobial resistance of staphylococci in canine pyoderma. Vet. Microbiol..

[12] Yoo JH, Yoon JW, Lee SY, Park HM (2010). High prevalence of fluoroquinolone- and methicillin-resistant *Staphylococcus **pseudintermedius* isolates from canine pyoderma and otitis externa in veterinary teaching hospital. J. Microbiol. Biotechnol..

[13] Bryan J (2012). Treatment outcome of dogs with meticillin-resistant and meticillin-susceptible *Staphylococcus **pseudintermedius* pyoderma. Vet. Dermatol..

[14] Wang Y (2012). Methicillin-resistant *Staphylococcus **pseudintermedius* isolated from canine pyoderma in North China. J. Appl. Microbiol..

[15] Beck KM, Waisglass SE, Dick HLN, Weese JS (2012). Prevalence of meticillin-resistant *Staphylococcus **pseudintermedius* (MRSP) from skin and carriage sites of dogs after treatment of their meticillin-resistant or meticillin-sensitive staphylococcal pyoderma. Vet. Dermatol..

[16] Bugden D (2012). Identification and antibiotic susceptibility of bacterial isolates from dogs with otitis externa in Australia. Aust. Vet. J..

[17] Dziva F (2015). First identification of methicillin-resistant *Staphylococcus **pseudintermedius* strains among coagulase-positive staphylococci isolated from dogs with otitis externa in Trinidad, West Indies. Infect. Ecol. Epidemiol..

[18] Zur G, Lifshitz B, Bdolah-Abram T (2011). The association between the signalment, common causes of canine otitis externa and pathogens. J. Small. Anim. Pract..

[19] Sim JXF, Khazandi M, Chan WY, Trott DJ, Deo P (2019). Antimicrobial activity of thyme oil, oregano oil, thymol and carvacrol against sensitive and resistant microbial isolates from dogs with otitis externa. Vet. Dermatol..

[20] Scherer C (2018). Frequency and antimicrobial susceptibility of *Staphylococcus **pseudintermedius* in dogs with otitis externa. Cienc. Rural..

[21] Rynhoud H (2021). Epidemiology of methicillin resistant *Staphylococcus* species carriage in companion animals in the Greater Brisbane Area, Australia. Res. Vet. Sci..

[22] Lo W-T (2010). Comparative molecular analysis of meticillin-resistant *Staphylococcus aureus* isolates from children with atopic dermatitis and healthy subjects in Taiwan. Br. J. Dermatol..

[23] Sai N, Laurent C, Strale H, Denis O, Byl B (2015). Efficacy of the decolonization of methicillin-resistant *Staphylococcus aureus* carriers in clinical practice. Antimicrob. Resist. Infect. Control.

[24] Rodrigues Hoffmann A (2014). The skin microbiome in healthy and allergic dogs. PLoS ONE.

[25] Bradley CW (2016). Longitudinal evaluation of the skin microbiome and association with microenvironment and treatment in canine atopic dermatitis. J. Invest. Dermatol..

[26] Apostolopoulos N (2021). Description and comparison of the skin and ear canal microbiota of non-allergic and allergic German shepherd dogs using next generation sequencing. PLoS ONE.

[27] Chermprapai S (2019). The bacterial and fungal microbiome of the skin of healthy dogs and dogs with atopic dermatitis and the impact of topical antimicrobial therapy, an exploratory study. Vet. Microbiol..

[28] Tress B (2017). Bacterial microbiome of the nose of healthy dogs and dogs with nasal disease. PLoS ONE.

[29] Cuscó A, Sánchez A, Altet L, Ferrer L, Francino O (2017). Individual signatures define canine skin microbiota composition and variability. Front. Vet. Sci..

[30] Shi B, Leung DYM, Taylor PA, Li H (2018). Methicillin-resistant *Staphylococcus aureus* colonization is associated with decreased skin commensal bacteria in atopic dermatitis. J. Invest. Dermatol..

[31] Hyejin K (2015). A double-blind, placebo controlled-trial of a probiotic strain *Lactobacillus **sakei* probio-65 for the prevention of canine atopic dermatitis. J. Microbiol. Biotechnol..

[32] Hensel P, Santoro D, Favrot C, Hill P, Griffin C (2015). Canine atopic dermatitis: Detailed guidelines for diagnosis and allergen identification. BMC Vet. Res..

[33] Kumar S, Hussain K, Sharma R, Chhibber S, Sharma N (2014). Prevalence of canine otitis externa in Jammu. J. Anim. Res..

[34] Harvey ND, Shaw SC, Craigon PJ, Blott SC, England GCW (2019). Environmental risk factors for canine atopic dermatitis: A retrospective large-scale study in Labrador and golden retrievers. Vet. Dermatol..

[35] Nødtvedt A, Egenvall A, Bergval K, Hedhammar Å (2006). Incidence of and risk factors for atopic dermatitis in a Swedish population of insured dogs. Vet. Rec..

[36] O’Neill DG (2021). Frequency and predisposing factors for canine otitis externa in the UK—A primary veterinary care epidemiological view. Canine Med. Genet..

[37] O'Neill DG, Coulson NR, Church DB, Brodbelt DC (2017). Demography and disorders of German shepherd dogs under primary veterinary care in the UK. Canine Genet. Epidemiol..

[38] Olivry T (2010). Treatment of canine atopic dermatitis: 2010 clinical practice guidelines from the International Task Force on Canine Atopic Dermatitis. Vet. Dermatol..

[39] Bajwa J (2019). Canine otitis externa—Treatment and complications. Can. Vet. J..

[40] Flemer B (2018). The oral microbiota in colorectal cancer is distinctive and predictive. Gut.

[41] Walters WA, Xu Z, Knight R (2014). Meta-analyses of human gut microbes associated with obesity and IBD. FEBS Lett..

[42] Zeller G (2014). Potential of fecal microbiota for early-stage detection of colorectal cancer. Mol. Syst. Biol..

[43] Qin N (2014). Alterations of the human gut microbiome in liver cirrhosis. Nature.

[44] Zou H, Hastie T (2005). Regularization and variable selection via the elastic net. J. R. Stat. Soc. Ser. B (Stat. Methodol.).

[45] Kawakami T (2010). Antimicrobial susceptibility and methicillin resistance in *Staphylococcus **pseudintermedius* and *Staphylococcus **schleiferi* subsp. *coagulans* isolated from dogs with pyoderma in Japan. J. Vet. Med. Sci..

[46] Siak M (2014). Characterization of meticillin-resistant and meticillin-susceptible isolates of *Staphylococcus **pseudintermedius* from cases of canine pyoderma in Australia. J. Med. Microbiol..

[47] Kania SA (2004). Methicillin resistance of staphylococci isolated from the skin of dogs with pyoderma. Am. J. Vet. Res..

[48] Isaiah A (2017). Characterization of the nasal and oral microbiota of detection dogs. PLoS ONE.

[49] Masiuk H, Wcisłek A, Jursa-Kulesza J (2021). Determination of nasal carriage and skin colonization, antimicrobial susceptibility and genetic relatedness of *Staphylococcus aureus* isolated from patients with atopic dermatitis in Szczecin, Poland. BMC Infect. Dis..

[50] Alsterholm M (2017). Variation in *Staphylococcus aureus* colonization in relation to disease severity in adults with atopic dermatitis during a five-month follow-up. Acta Derm. Venereol..

[51] Yao H (2022). Absolute abundance values reveal microbial shifts and co-occurrence patterns during gut microbiota fermentation of dietary fibres in vitro. Food Hydrocoll..

[52] Engelbrektson A (2010). Experimental factors affecting PCR-based estimates of microbial species richness and evenness. ISME J..

[53] Bolyen E (2019). Reproducible, interactive, scalable and extensible microbiome data science using QIIME 2. Nat. Biotechnol..

[54] Callahan BJ (2016). DADA2: High-resolution sample inference from Illumina amplicon data. Nat. Methods..

[55] Quast C (2013). The SILVA ribosomal RNA gene database project: Improved data processing and web-based tools. Nucleic Acids Res..

[56] Microsoft 365. *Microsoft Excel*. https://www.microsoft.com/en-au/microsoft-365/excel?legRedir=true&CorrelationId=8ebc73df-5955-4130-b367-499fea70f562&rtc=1 (2022).

[57] Yan, L. *ggvenn: Draw Venn Diagram by 'ggplot2'*. https://CRAN.R-project.org/package=ggvenn (2021).

[58] Gehlenborg, N. *UpSetR: A More Scalable Alternative to Venn and Euler Diagrams for Visualizing Intersecting Sets*. https://CRAN.R-project.org/package=UpSetR (2019).

[59] Prabhakaran, S. *InformationValue: Performance Analysis and Companion Functions for Binary Classification Models*. https://CRAN.R-project.org/package=InformationValue (2016).

[60] Kuhn, M. *et al. Classification and Regression Training: Package 'Caret'*. https://cran.r-project.org/web/packages/caret/caret.pdf (2022).

[61] Hosmer, D. W., Lemeshow, S. & Sturdivant, R. X. *Applied Logistic Regression* (eds. Hosmer, D.W., Lemeshow, S., & Sturdivant, R.X.). 153–225 (Wiley, 2013).

[62] Kuhn, M. *caret: Classification and Regression Training*. https://CRAN.R-project.org/package=caret (2021).

[63] Larsen, K. *Information: Data Exploration with Information Theory (Weight-of-Evidence and Information Value)*. https://CRAN.R-project.org/package=Information (2016).

[64] Hamner, B. & Frasco, M. *Metrics: Evaluation Metrics for Machine Learning*. https://CRAN.R-project.org/package=Metrics (2018).

[65] Friedman JH, Hastie T, Tibshirani R (2010). Regularization paths for generalized linear models via coordinate descent. J. Stat. Softw..

[66] Csardi G, Nepusz T (2006). The igraph software package for complex network research. Int. J. Complex Syst..

[67] McMurdie PJ, Holmes S (2013). phyloseq: An R package for reproducible interactive analysis and graphics of microbiome census data. PLoS ONE.

