# Supplementary material for: Nasal microbiota profiles in shelter dogs with dermatological conditions carrying methicillin-resistant and methicillin-sensitive Staphylococcus species
Source: Sci Rep. 2023 Mar 24;13:4844. doi: 10.1038/s41598-023-31385-2 (PMC10039040; doi:10.1038/s41598-023-31385-2)
Supplement: Supplementary file 1 — Supplementary Information. [file 41598_2023_31385_MOESM1_ESM.docx]

**Nasal microbiota profiles in shelter dogs with dermatological conditions carrying methicillin-resistant and methicillin-sensitive *Staphylococcus* species**

Sara Horsman^1*^, Erika Meler^1^, Deirdre Mikkelsen^2*^, John Mallyon^1^, Hong Yao^3^, Ricardo J. Soares Magalhães^1,4^ and Justine S. Gibson^1^

**Complete postal address(es) of affiliations**

^1^School of Veterinary Science, The University of Queensland, Gatton 4343, Queensland, Australia

^2^School of Agriculture and Food Sciences, The University of Queensland, St Lucia 4072, Queensland, Australia

^3^Centre for Nutrition and Food Sciences, Queensland Alliance for Agriculture and Food Innovation, The University of Queensland, St Lucia 4072, Queensland, Australia

^4^Children Health and Environment Program, Child Health Research Centre, The University of Queensland, South Brisbane 4101, Queensland, Australia

1. **Supplementary information**
   1. **Supplementary results**
      1. **Nasal carriage of methicillin-resistant and -sensitive *Staphylococcus* spp.**

Of the 70 dogs, 41 had only one *Staphylococcus* spp. isolated, 16 had two different *Staphylococcus* spp., five dogs had three different *Staphylococcus* spp. isolated, and eight dogs were negative for *Staphylococcus* or culture negative for all bacteria regardless of the number of samples per dog. The matrix assisted laser desorption ionisation – time of flight mass spectrometry (MALDI-TOF MS) results indicated that six of the staphylococci isolates were only acceptable at the genus level and these isolates will be referred to as suspect staphylococci species (as required). These included: one methicillin-sensitive *S*. *pseudintermedius* (MSSP), three methicillin-sensitive S. epidermis, one methicillin-sensitive S. simulans, and the only methicillin-sensitive S. felis.

Overall, at any time of the sampling, 10 dogs were culture positive for methicillin-resistant *S*. *pseudintermedius* (MRSP), 43 were culture positive for MSSP, two for methicillin-resistant *S*. *aureus* (MRSA), seven for methicillin-sensitive *S*. *aureus* (MSSA), 10 for methicillin-resistant coagulase-negative *Staphylococcus* spp. (MR-CoNS) and 22 being cultured positive for methicillin-sensitive coagulase-negative *Staphylococcus* spp. (MS-CoNS). Additionally, 22 dogs were culture negative for staphylococci, and 12 dogs were culture negative for all bacteria on the selective agar plates throughout the sampling period.

Methicillin-resistant *S*. *pseudintermedius* was isolated from 10 dogs (5%; 10/186 nasal samples) with 8% (3/39) of nasal samples from dogs with dermatological conditions and 5% (7/144) were from dogs without those conditions (Table S1). MSSP was the most frequently isolated *Staphylococcus* spp*.* and was isolated from 40 dogs (40%; 74/186) with 46% (18/39) of samples from dogs with dermatological conditions and 39% (56/144) from dogs without those conditions (Table S1). MRSA was isolated from two dogs, one with and one without dermatological conditions (3%; 5/186); 10% (4/39) of samples belonged to the dog with dermatological conditions (Table S1). MSSA was isolated from seven dogs with 3% (1/39) of samples from dogs with dermatological conditions compared to 10% (15/144) of samples from dogs without these conditions (Table S1). Overall, MR-CoNS were isolated from 10 dogs (6%; 12/186 nasal samples) and MS-CoNS were isolated from 22 dogs (13%; 25/186 nasal samples). For the total number of nasal isolates cultured from dogs with or without dermatological conditions, refer to Table S1.

**Table S1** Nasal samples corresponding to dogs with and without dermatological conditions and their methicillin-resistant and -sensitive Staphylococcus spp. nasal carriage culture results

| **Nasal samples from** | | **Nasal carriage: bacterial culture results** | | | | | | | | |
| --- | --- | --- | --- | --- | --- | --- | --- | --- | --- | --- |
|  |  | **MRSP** | **MSSP** | **MRSA** | **MSSA** | **MR-CoNS^a^** | **MS-CoNS^b^** | **Culture negative for *Staphylococcus*** | **Culture negative for all bacteria** | **Total nasal samples per group** |
| **Dogs with dermatological conditions (N = 16)** | **n (%)** | 3 (8) | 18 (46) | 4 (10) | 1 (3) | 1 (3) | 4 (10) | 5 (13) | 3 (8) | 39 (21) |
| **Dogs without dermatological conditions (N = 52)** | **n (%)** | 7 (5) | 56 (39) | 1 (1) | 15 (10) | 11 (8) | 20 (14) | 21 (14) | 13 (9) | 144 (77) |
| **Dogs with no medical history (N = 2)** | **n (%)** | 0 (0) | 0 (0) | 0 (0) | 0 (0) | 0 (0) | 1 (33) | 2 (67) | 0 (0) | 3 (2) |
| **Total number of nasal isolates (N = 186)** | **n (%)** | 10 (5) | 74 (40) | 5 (3) | 16 (9) | 12 (6) | 25 (13) | 28 (15) | 16 (9) | 186 (100) |

The percentages are based on the number of isolates per the total number of nasal samples in the last column. For instance, 8% (3/39) of nasal samples from dogs with dermatological conditions were MRSP-positive.

MRSP = methicillin-resistant *S*. *pseudintermedius*; MSSP = methicillin-sensitive *S*. *pseudintermedius*; MRSA = methicillin-resistant *S*. *aureus*; MSSA = methicillin-sensitive *S*. *aureus*; MR-CoNS = methicillin-resistant coagulase-negative staphylococci; MS-CoNS = methicillin-sensitive coagulase-negative staphylococci.

^a^MR-CoNS species: *S*. *epidermis* (n = 7); *S*. *haemolyticus* (n = 3); *S*. *pasteuri* (n = 1); and *S*. *lentus* (n = 1).

^b^MS-CoNS species: *S*. *epidermis* (n =15); *S*. *simulans* (n =4); *S*. *capitis* (n = 2); *S*. *haemolyticus*; *S*. *hominis*; *S*. *warneri*; and *S*. *felis* (n = 1 each).

- - 1. **Antimicrobial susceptibility results of** **nasal methicillin-resistant and -sensitive *Staphylococcus* spp. isolates**

For the overall antimicrobial susceptibility results for dogs with and without dermatological conditions, refer to Table S2 and Table S3, respectively. All MRS spp. isolates were multidrug resistant (MDR) (100%; 27/27)^1^. All methicillin-resistant isolates were positive for the *mecA* gene and only one MSSP isolate was oxacillin-resistant and *mecA* negative. Three MSSP isolates from dogs with dermatological conditions were MDR. A MS-CoNS (*S*. *haemolyticus*) isolate from a dog without dermatological conditions was also MDR^1^. No MSSA were MDR.

**Table S2** Antimicrobial resistance profile of methicillin-resistant and -sensitive Staphylococcus spp. isolates cultured from 16 of the 68 shelter dogs with dermatological conditions (N = 30 nasal samples)

| **Isolates** | | **Antimicrobial drugs** | | | | | | | | | | | | | | |
| --- | --- | --- | --- | --- | --- | --- | --- | --- | --- | --- | --- | --- | --- | --- | --- | --- |
|  |  | **AK** | **AMC** | **C** | **CPD** | **DA** | **ENR** | **E** | **FOX** | **GEN** | **KF** | **OX** | **P** | **TE** | **SXT** | **MDR** |
| **MRSP  (N = 3)** | **n (%)** | 0  (0) | 1  (33) | 1  (33) | 3 (100) | 1  (33) | 1  (33) | 1  (33) | NT | 0  (0) | 1  (33) | 3 (100) | 3 (100) | 1  (33) | 1  (33) | 3 (100) |
| **MSSP  (N = 18)** | **n (%)** | 1  (6) | 0  (0) | 1  (6) | 1  (6) | 1  (6) | 3  (17) | 2  (11) | NT | 2  (11) | 1  (6) | 1  (6) | 10  (56) | 3  (17) | 0  (0) | 3  (17) |
| **MRSA**  **(N = 4)** | **n (%)** | 0  (0) | 4 (100) | 0  (0) | 4 (100) | 0  (0) | 4 (100) | 4 (100) | 4 (100) | 4 (100) | 4 (100) | NT | 4 (100) | 0  (0) | 0  (0) | 4 (100) |
| **MSSA**  **(N = 1)** | **n (%)** | 0  (0) | 0  (0) | 0  (0) | 0  (0) | 0  (0) | 0  (0) | 0  (0) | 0  (0) | 0  (0) | 0  (0) | NT | 1 (100) | 0  (0) | 0  (0) | 0  (0) |
| **MR-CoNS**  **(N = 1)** | **n (%)** | 0  (0) | 0  (0) | 0  (0) | 1 (100) | 0  (0) | 0  (0) | 0  (0) | 1 (100) | 0  (0) | 0  (0) | NT | 1 (100) | 0  (0) | 0  (0) | 1 (100) |
| **MS-CoNS**  **(N = 4)** | **n (%)** | 0  (0) | 0  (0) | 0  (0) | 0  (0) | 0  (0) | 0  (0) | 1  (25) | 0  (0) | 0  (0) | 0  (0) | NT | 3  (75) | 1  (25) | 0  (0) | 0  (0) |

Antimicrobial resistance included both intermediate resistance and resistance classification. Aminoglycosides: amikacin (AK 30 µg), gentamicin (GEN 10 µg); Amphenicols: chloramphenicol (C 30 µg); Cephalosporins: cefoxitin (FOX 30 µg), cefpodoxime (CPD 10 µg) and cephalothin (KF 30 µg); Fluoroquinolone: enrofloxacin (ENR 5 µg); Lincomycin: clindamycin (DA 2 µg); Macrolide: erythromycin (E 15 µg); Penicillins (+/- β-lactamase inhibitors): amoxicillin/clavulanate (AMC 20/10 µg), oxacillin (OX 1 µg) and penicillin (P 10 units); Tetracyclines: tetracycline (TE 30 µg); Sulfonamide: trimethoprim-sulfamethoxazole (SXT 1.25/23.75 µg); MDR = multidrug resistant; NT = not tested.

MRSP = methicillin-resistant *S*. *pseudintermedius*; MSSP = methicillin-sensitive *S*. *pseudintermedius*; MRSA = methicillin-resistant *S*. *aureus*; MSSA = methicillin-sensitive *S*. *aureus*; MR-CoNS = methicillin-resistant coagulase-negative staphylococci; MS-CoNS = methicillin-sensitive coagulase-negative staphylococci.

**Table S3** Antimicrobial resistance profile of methicillin-resistant and -sensitive *Staphylococcus* spp. isolates cultured from 52 of the 68 shelter dogs without dermatological conditions (N = 111 nasal samples)

| **Isolates** | | **Antimicrobial drugs** | | | | | | | | | | | | | | |
| --- | --- | --- | --- | --- | --- | --- | --- | --- | --- | --- | --- | --- | --- | --- | --- | --- |
|  |  | **AK** | **AMC** | **C** | **CPD** | **DA** | **ENR** | **E** | **FOX** | **GEN** | **KF** | **OX** | **P** | **TE** | **SXT** | **MDR** |
| **MRSP  (N = 7)** | **n (%)** | 0  (0) | 5  (71) | 5 (71) | 7  (100) | 5  (71) | 5  (71) | 5  (71) | NT | 1  (14) | 5  (71) | 7 (100) | 7  (100) | 5  (71) | 5  (71) | 7 (100) |
| **MSSP  (N = 56)** | **n (%)** | 0  (0) | 0  (0) | 0  (0) | 0  (0) | 1  (2) | 0  (0) | 2  (4) | NT | 0  (0) | 0  (0) | 0  (0) | 44 (79) | 7  (13) | 1  (2) | 0  (0) |
| **MRSA  (N = 1)** | **n (%)** | 0  (0) | 1  (100) | 0  (0) | 1  (100) | 0  (0) | 0  (0) | 0  (0) | 1  (100) | 0  (0) | 1 (100) | NT | 1  (100) | 0  (0) | 0  (0) | 1 (100) |
| **MSSA  (N = 15)** | **n (%)** | 0  (0) | 0  (0) | 0  (0) | 2  (13) | 0  (0) | 0  (0) | 0  (0) | 0  (0) | 0  (0) | 0  (0) | NT | 9  (60) | 0  (0) | 0  (0) | 0  (0) |
| **MR-CoNS  (N = 11)** | **n (%)** | 2  (18) | 4  (36) | 1 (9) | 10  (91) | 9  (82) | 3  (27) | 6  (55) | 11  (100) | 3  (27) | 3  (27) | NT | 11  (100) | 9  (82) | 4  (36) | 11 (100) |
| **MS-CoNS  (N = 20)^a^** | **n (%)** | 0  (0) | 0  (0) | 0  (0) | 0  (0) | 0  (0) | 0  (0) | 3  (15) | 0  (0) | 0  (0) | 0  (0) | NT | 13 (65) | 9  (45) | 1  (5) | 1  (5) |

Antimicrobial resistance included both intermediate resistance and resistance classification. Aminoglycosides: amikacin (AK 30 µg), gentamicin (GEN 10 µg); Amphenicols: chloramphenicol (C 30 µg); Cephalosporins: cefoxitin (FOX 30 µg), cefpodoxime (CPD 10 µg) and cephalothin (KF 30 µg); Fluoroquinolone: enrofloxacin (ENR 5 µg); Lincomycin: clindamycin (DA 2 µg); Macrolide: erythromycin (E 15 µg); Penicillins (+/- β-lactamase inhibitors): amoxicillin/clavulanate (AMC 20/10 µg), oxacillin (OX 1 µg) and penicillin (P 10 units); Tetracyclines: tetracycline (TE 30 µg); Sulfonamide: trimethoprim-sulfamethoxazole (SXT 1.25/23.75 µg); MDR = multidrug resistant; NT = not tested.

MRSP = methicillin-resistant *S*. *pseudintermedius*; MSSP = methicillin-sensitive *S*. *pseudintermedius*; MRSA = methicillin-resistant *S*. *aureus*; MSSA = methicillin-sensitive *S*. *aureus*; MR-CoNS = methicillin-resistant coagulase-negative staphylococci; MS-CoNS = methicillin-sensitive coagulase-negative staphylococci.

^a^The MS-CoNS isolate was removed from this analysis due to the lack of available medical history data (refer to Table S1 above).

- - 1. **Risk factors associated with dogs with dermatological conditions in the animal shelter**
       1. **Univariable analysis**

For the univariable models, dogs with dermatological conditions had higher odds of being female [Odds Ratio (OR): 3.46 (95% CI: 1.38-8.62); *p=*0.008] and being present in the shelter two to seven days prior to the initial swab being taken compared to dogs without those conditions [OR: 3.23 (95% CI: 2.04-5.12); *p* ≤0.001] (Table S4). Additionally, dogs with dermatological conditions had higher odds of being from the humane officer seized (or surrendered) dog population [OR: 2.50 (95% CI: 1.53-4.07); *p* ≤0.001]. Whereas there were lowered odds for dogs with those conditions for the owner surrendered dog population [OR: 0.42 (95% CI: 0.20-0.83); *p=*0.019]. If dogs had dermatological conditions, they were at higher odds of having a long length of stay at the shelter and were more likely to be held in the veterinary clinic’s dog holding [OR: 3.81 (95% CI: 2.57-5.66); *p* ≤0.001] (Table S4). MRSA was the only statistically significant nasal carriage in dogs with dermatological conditions at the shelter [OR: 17.33 (95% CI: 5.04-59.65); *p* ≤0.001] (Table S4). Dogs with dermatological conditions had higher odds of being treated with antimicrobials than dogs without those conditions [OR: 16.12 (95% CI: 6.79-38.30); *p* ≤0.001]. Dogs had lowered odds of having dermatological conditions if they were originally located to the north and west of Brisbane compared to dogs without dermatological conditions [OR: 0.47 (95% CI: 0.38-0.58); *p* ≤0.001 and OR: 0.54 (95% CI: 0.38-0.77); *p=*0.001, respectively]. If dogs were present at another animal shelter before entering the sampling shelter, there were significantly lower odds of dogs having dermatological conditions (Table S4). Age, breed size, and neuter status were not statistically significant for dogs with these conditions. For the complete results of the univariable models, refer to Table S4.

**Table S4** Univariable analysis of the risk factors associated with shelter dogs with dermatological conditions (N = 183 nasal samples)

| **Variables** | **Univariable analysis** | | | | **Variables** | **Univariable analysis** | | | |
| --- | --- | --- | --- | --- | --- | --- | --- | --- | --- |
|  | **n** | **Odds ratio  (95% CI)** | ***p*-value** | **Overall *p*-value** |  | **n** | **Odds ratio  (95% CI)** | ***p*-value** | **Overall *p*-value** |
| **Age (years)  (N = 181)** |  |  |  |  | **Dog population** |  |  |  |  |
| ≤3 | 119 | Reference |  | 0.415 | Strays | 90 | Reference |  | ≤0.001 |
| >3-6 | 30 | 0.52 (0.18 - 1.56) | 0.245 |  | Owner surrendered | 36 | 0.42 (0.20 - 0.86) | 0.019 |  |
| ≥6 | 32 | 1.13 (0.89 - 1.45) | 0.313 |  | Humane officer seized | 57 | 2.50 (1.53 - 4.07) | ≤0.001 |  |
| **Sex** |  |  |  |  | **Original location of dogs** |  |  |  |  |
| Male | 69 | Reference |  | 0.008 | Brisbane | 33 | Reference |  | ≤0.001 |
| Female | 114 | 3.46 (1.38 - 8.62) | 0.008 |  | North of Brisbane | 41 | 0.47 (0.38 - 0.58) | ≤0.001 |  |
| **Breed size** |  |  |  |  | South of Brisbane | 25 | 0.73 (0.20 - 2.58) | 0.621 |  |
| Small | 19 | Reference |  | 0.489 | West of Brisbane | 84 | 0.54 (0.38 - 0.77) | 0.001 |  |
| Medium | 111 | 1.09 (0.35 - 3.35) | 0.880 |  | **Previous shelter location** |  |  |  |  |
| Large | 53 | 0.87 (0.31 - 2.42) | 0.793 |  | Shelter one (sampling shelter) | 120 | Reference |  | ≤0.001 |
| **Neuter status  (N = 167)** |  |  |  |  | Shelter two | 28 | 0.30 (0.20 - 0.46) | ≤0.001 |  |
| Entire | 12 | Reference |  | 0.351 | Shelter three | 35 | 0.15 (0.07 - 0.32) | ≤0.001 |  |
| Neutered | 155 | 1.30 (0.75 - 2.26) | 0.351 |  | **Antimicrobial usage** |  |  |  |  |
| **Days in shelter prior to initial swab being taken** |  |  |  |  | No | 146 | Reference |  | ≤0.001 |
| ≤1 | 119 | Reference |  | ≤0.001 | Yes | 37 | 16.12 (6.79 - 38.30) | ≤0.001 |  |
| 2-7 | 52 | 3.23 (2.04 - 5.12) | ≤0.001 |  |  |  |  |  |  |
| >7 | 12 | 1.12 (0.66 - 1.92) | 0.673 |  |  |  |  |  |  |

The total number of nasal samples equalled 183 unless otherwise specified in the table. n = the number of individual samples per variable.

**Table S4 continued.**

| **Variables** | **Univariable analysis** | | | | **Variables** | **Univariable analysis** | | | |
| --- | --- | --- | --- | --- | --- | --- | --- | --- | --- |
|  | **n** | **Odds ratio  (95% CI)** | ***p*-value** | **Overall *p*-value** |  | **n** | **Odds ratio  (95% CI)** | ***p*-value** | **Overall *p*-value** |
| **Sampling location within the shelter** |  |  |  |  | **Nasal carriage** |  |  |  |  |
| Shelters’ veterinary clinic | 89 | Reference |  | ≤0.001 | Culture negative for all bacteria | 16 | Reference |  | ≤0.001 |
| Shelters’ veterinary clinic dog holding | 19 | 3.81 (2.57 - 5.66) | ≤0.001 |  | Staphylococci culture negative | 26 | 1.03 (0.17 - 6.31) | 0.973 |  |
| Shelters’ dog holdings | 63 | 1.10 (0.66 - 1.83) | 0.710 |  | MRSP | 10 | 1.86 (0.15 - 22.86) | 0.629 |  |
| Shelters’ adoption centre | 12 | NA |  |  | MSSP | 74 | 1.39 (0.40 - 4.82) | 0.601 |  |
| **Length of stay (days)** |  |  |  |  | MRSA | 5 | 17.33 (5.04 - 59.65) | ≤0.001 |  |
| 0-7 | 67 | Reference |  | ≤0.001 | MSSA | 16 | 0.29 (0.03 - 2.92) | 0.293 |  |
| 8-14 | 23 | 3.75 (2.07 - 6.78) | ≤0.001 |  | MR-CoNS | 12 | 0.39 (0.07 - 2.12) | 0.278 |  |
| 15-21 | 45 | 2.77 (1.67 - 4.62) | ≤0.001 |  | MS-CoNS | 24 | 0.87 (0.23 - 3.21) | 0.830 |  |
| ≥22 | 48 | 3.53 (1.80 - 6.93) | ≤0.001 |  |  |  |  |  |  |

For the dogs without any signalment data available, those cells were left blank in the spreadsheet used to run the Bernoulli model. For the sampling location within the shelter, the shelter’s dog adoption centre was omitted from the model as it predicts failure perfectly. The total number of nasal samples equalled 183 unless otherwise specified in the table. n = the number of individual samples per variable.

MRSP = methicillin-resistant *S*. *pseudintermedius*; MSSP = methicillin-sensitive *S*. *pseudintermedius*; MRSA = methicillin-resistant *S*. *aureus*; MSSA = methicillin-sensitive *S*. *aureus*; MR-CoNS = methicillin-resistant coagulase-negative staphylococci; MS-CoNS = methicillin-sensitive coagulase-negative staphylococci.

- - 1. **Comparison of the nasal microbiota from dogs with and without dermatological conditions**
       1. **Nasal carriage included in the microbiota analyses**

**Table S5** Staphylococcus spp. nasal carriage of dogs with and without dermatological conditions isolated based on the baseline and follow-up sampling

| **Bacterial nasal culture results** | **Dogs without dermatological conditions** | | **Dogs with dermatological conditions** | |
| --- | --- | --- | --- | --- |
|  | **Baseline samples** | **Follow-up samples** | **Baseline samples** | **Follow-up**  **samples** |
| **Culture negative for all bacteria** | 2 | 0 | 0 | 1 |
| **Staphylococci culture negative** | 6 | 2 | 1 | 0 |
| **MRSP** | 2 | 1 | 2 | 0 |
| **MSSP** | 11 | 6 | 2 | 5 |
| **MRSA** | 1 | 0 | 0 | 1 |
| **MSSA** | 2 | 0 | 1 | 0 |
| **MR-CoNS** | 1 | 1 | 0 | 0 |
| **MS-CoNS** | 3 | 1 | 0 | 0 |
| **Total (N = 52)** | **28** | **11** | **6** | **7** |

MRSP = methicillin-resistant *S*. *pseudintermedius*; MSSP = methicillin-sensitive
*S*. *pseudintermedius*; MRSA = methicillin-resistant *S*. *aureus*; MSSA = methicillin-sensitive
*S*. *aureus*; MR-CoNS = methicillin-resistant coagulase-negative staphylococci; MS-CoNS = methicillin-sensitive coagulase-negative staphylococci.

- - - 1. **Alpha and beta diversities of nasal carriage and antimicrobial usage**

The alpha diversity plots for nasal carriage of MRS spp., MSS spp., and staphylococci culture negative/culture negative for all bacteria (negative) were not significantly different for dogs with and without dermatological conditions (Figure S1). Figure S2a also showed that there were no significant differences in alpha diversities between dogs with and without dermatological conditions that were treated using any antimicrobials (i.e., topical, or oral antimicrobials). No clustering was observed in the beta diversity of dogs with and without dermatological conditions treated using any antimicrobials (Figure S2b).


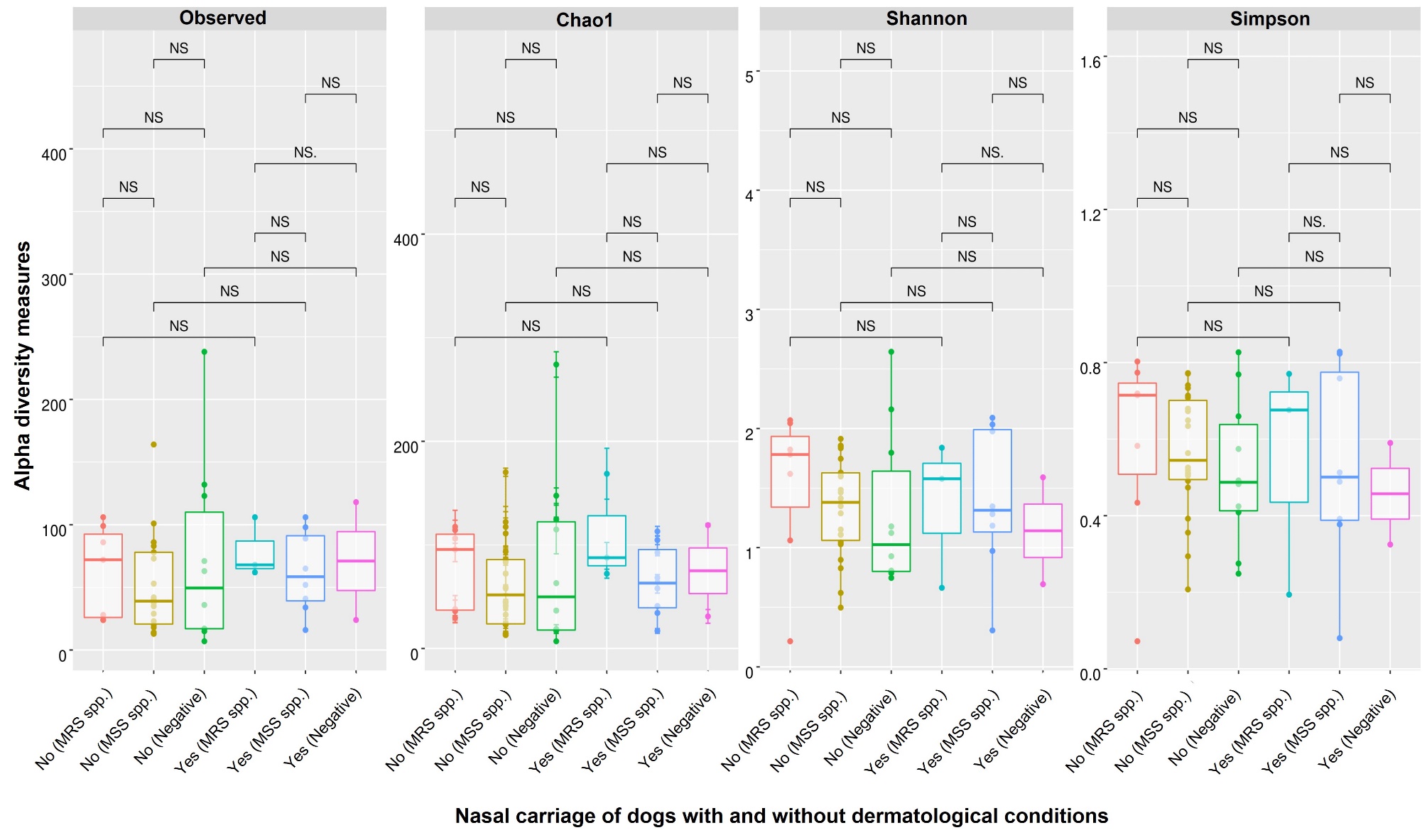


**Figure S1** Alpha diversity plots of nasal samples displaying the observed richness, Chao1, Shannon Index, and Simpson’s Index for dogs with (yes) and without (no) dermatological conditions that were culture positive for MRS spp. and MSS spp. or staphylococci culture negative/culture negative for all bacteria (negative) over the study period using Wilcoxon rank-sum test significance (p >0.05 = NS (not significant).


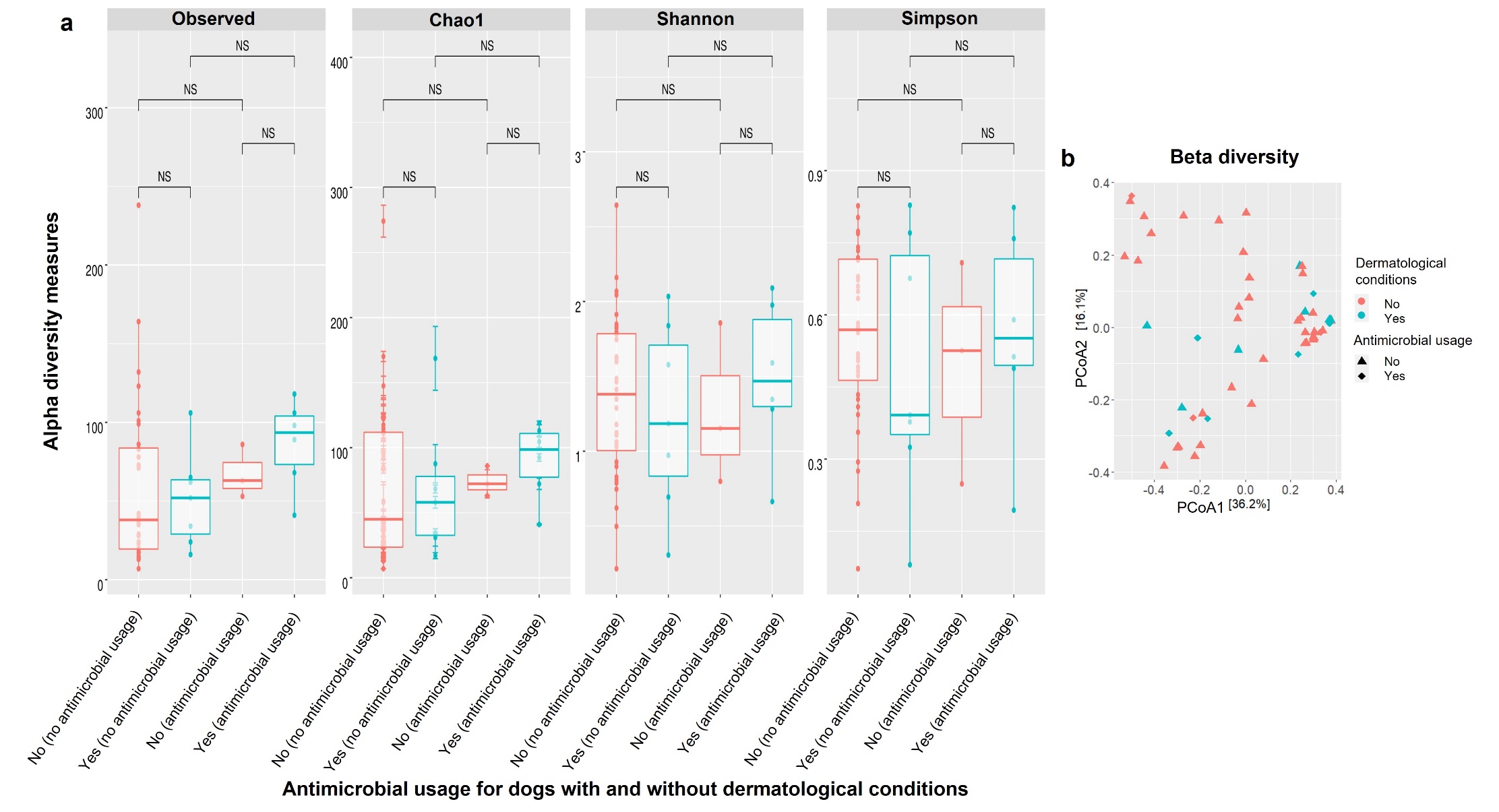


**Figure S2** Microbiota analysis of nasal samples from dogs with (yes) and without (no) dermatological conditions displaying the **(a)** alpha diversity plots of observed richness, Chao1, Shannon Index, and Simpson’s Index for dogs with and without antimicrobial usage over the study period using Wilcoxon rank-sum test significance (p >0.05 = NS (not significant); and **(b)** PCoA plots with Bray-Curtis displaying the amplicon sequence variants (ASVs) from the nasal samples of dogs with (yes) and without (no) antimicrobial usage.

- - - 1. **Core microbiota for the *Staphylococcus* spp. nasal carriage status and identification of shared and core genera for all analyses**

To interpret the UpSet plot in Figure S3, the turquoise left side bars represent the counts of genera per dermatological condition and nasal carriage. For instance, dogs without dermatological conditions carrying MSSP (No – MSSP) had 10 identified genera. The violet-red column graph displays the number of genera belonging to the different groups. The purple-blue dotted lines represent the intersecting of the data. The dots indicate the individual nasal group and the lines indicate that more than one group has those shared genera which are equivalent to an area in a Venn diagram^2^. If only one dot is present without any intersecting lines, then X number of genera were unique to that group. As an example, dogs with dermatological conditions carrying MRSA had four genera unique to that group, including *Bacillus*, *Collinsella*, *Enterococcus*, and *Romboutsia*, represented by the single dot in the second column from the left-hand side of the plot (Figure S3). Dogs without those conditions that were culture negative for *Staphylococcus* spp. also had four genera unique to that group (dot in the first column from the left in Figure S3). These genera included *Acidibacter*, *Bacteroides*, *Faecalibacterium*, *Serratia*, and the unclassified Solirubrobacterales (genera code 67-14).

For all genera identified in the core microbiota analyses and whether they were classified as pan (genera present in some but not all groups), core (genera present in all groups), or unique (genera present in only one group) from dogs with and without dermatological conditions, antimicrobial usage, and nasal carriage, refer to Tables S6 to S8 below.

**
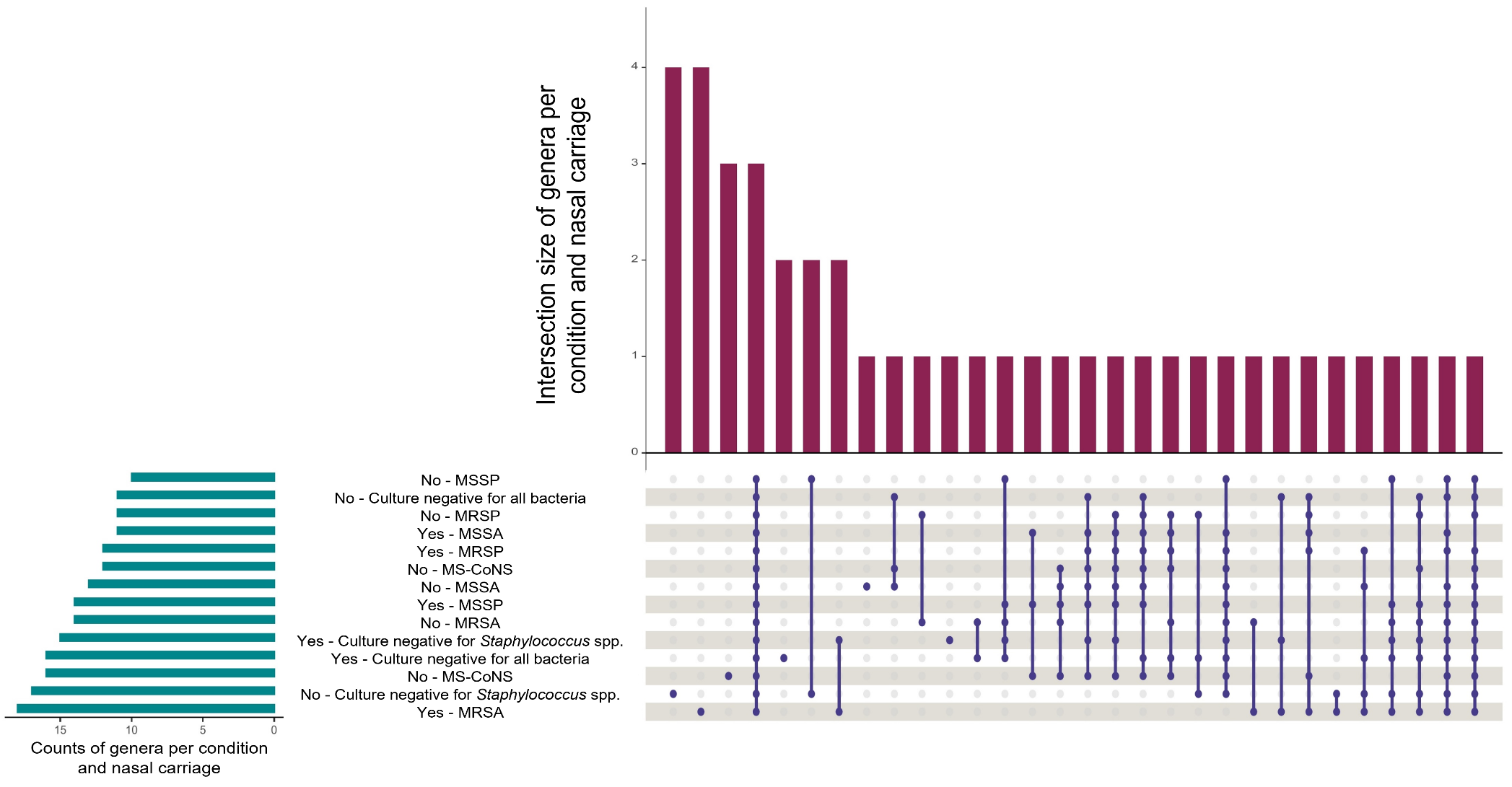
**

**Figure S3** UpSet plot displaying the number of shared and unique genera for dogs with (yes) and without (no) dermatological conditions and the corresponding nasal carriage status. For instance, No – MSSP referred to dogs without dermatological conditions that cultured MSSP from the nares.

MRSP = methicillin-resistant *S*. *pseudintermedius*; MSSP = methicillin-sensitive *S*. *pseudintermedius*; MRSA = methicillin-resistant
*S*. *aureus*; MSSA = methicillin-sensitive *S*. *aureus*; MR-CoNS = methicillin-resistant coagulase-negative staphylococci; MS-CoNS = methicillin-sensitive coagulase-negative staphylococci.

**Table S6** Core microbiota identifying the core, unique and pan genera of dogs with (yes) and without (no) dermatological conditions

| **Genera** | **Type** | **Group** | **Genera** | **Type** | **Group** |
| --- | --- | --- | --- | --- | --- |
| [Ruminococcus]_  gnavus_group | Unique | No | *Gracilibacteria* | Core | Yes and No |
| Solirubrobacterales (genera code: 67-14) | Unique | Yes | *Granulicatella* | Core | Yes and No |
| *Acidibacter* | Unique | Yes | *Helcococcus* | Core | Yes and No |
| *Aspergillus* | Unique | No | JGI_0000069-P22 | Core | Yes and No |
| *Bacillus* | Unique | No | *Klebsiella* | Unique | No |
| *Bacteroides* | Unique | Yes | *Lactobacillus* | Unique | No |
| *Bergeyella* | Unique | No | *Leifsonia* | Core | Yes and No |
| *Blautia* | Core | Yes and No | *Leucobacter* | Core | Yes and No |
| *Cellulomonas* | Core | Yes and No | *Massilia* | Core | Yes and No |
| Chloroplast | Unique | Yes | *Microbacterium* | Core | Yes and No |
| *Cladosporium* | Unique | Yes | *Moraxella* | Core | Yes and No |
| *Clostridium*_*sensu*_*stricto*_1 | Unique | No | *Pasteurella* | Unique | No |
| *Collinsella* | Unique | No | *Ralstonia* | Core | Yes and No |
| *Conchiformibius* | Unique | No | *Romboutsia* | Unique | No |
| *Craurococcus-Caldovatus* | Unique | No | *Rothia* | Unique | No |
| *Elizabethkingia* | Core | Yes and No | *Serratia* | Unique | Yes |
| *Embryophyta* | Core | Yes and No | *Staphylococcus* | Core | Yes and No |
| *Enterococcus* | Unique | No | *Streptococcus* | Core | Yes and No |
| *Escherichia-Shigella* | Unique | No | Unknown (other genera)^a^ | Core | Yes and No |
| Cardiobacteriaceae;  Uncultured genera | Core | Yes and No | *Ureaplasma* | Core | Yes and No |
| *Faecalibacterium* | Unique | Yes |  |  |  |

Within ‘group’, the ‘yes’ referred to dogs with dermatological conditions and the ‘no’ referred to dogs without dermatological conditions.

^a^Unknown should be interpreted lightly as it is not known which family or genera these microbes represent.

**Table S7** Core microbiota identifying the core, unique and pan genera of dogs with dermatological that were prescribed antimicrobials (Yes – antimicrobial usage) and not prescribed antimicrobials (Yes – No antimicrobials), and dogs without dermatological conditions that were prescribed antimicrobials (No – antimicrobial usage) and not prescribed antimicrobials (No – No antimicrobials)

| **Genera** | **Type** | **Group** | **Genera** | **Type** | **Group** |
| --- | --- | --- | --- | --- | --- |
| [Ruminococcus]_gnavus_group | Unique | No - No antimicrobial usage | *Craurococcus-Caldovatus* | Unique | No - No antimicrobial usage |
| Solirubrobacterales (genera code: 67-14) | Unique | Yes - No antimicrobial usage | *Elizabethkingia* | Pan | Yes - No antimicrobial usage and No - No antimicrobial usage |
| *Acidibacter* | Unique | Yes - No antimicrobial usage | *Embryophyta* | Pan | Yes - Antimicrobial usage and No - No antimicrobial usage |
| *Aspergillus* | Unique | No - Yes antimicrobial usage | *Enterococcus* | Unique | No - No antimicrobial usage |
| *Bacillus* | Unique | No - No antimicrobial usage | *Escherichia-Shigella* | Unique | No - Yes antimicrobial usage |
| *Bacteroides* | Unique | Yes - No antimicrobial usage | *Faecalibacterium* | Unique | Yes - No antimicrobial usage |
| *Bergeyella* | Unique | No - No antimicrobial usage | *Gracilibacteria* | Pan | Yes - No antimicrobial usage and No - No antimicrobial usage |
| *Blautia* | Pan | Yes - No antimicrobial usage and No - No antimicrobial usage | *Granulicatella* | Pan | Yes - No antimicrobial usage and No - No antimicrobial usage |
| Cardiobacteriaceae;  Uncultured genera | Pan | Yes - No antimicrobial usage and No - No antimicrobial usage | *Helcococcus* | Pan | Yes - No antimicrobial usage and No - No antimicrobial usage |
| *Cellulomonas* | Pan | Yes - Antimicrobial usage, No - No antimicrobial usage, and No - Yes antimicrobial usage | JGI_0000069-P22 | Pan | Yes - No antimicrobial usage, No - No antimicrobial usage, and No - Yes antimicrobial usage |
| Chloroplast | Unique | Yes - Antimicrobial usage | *Klebsiella* | Unique | No - Yes antimicrobial usage |
| *Cladosporium* | Unique | Yes - Antimicrobial usage | *Lactobacillus* | Unique | No - No antimicrobial usage |
| *Clostridium*_*sensu*_*stricto*_1 | Unique | No - No antimicrobial usage | *Leifsonia* | Pan | Yes - Antimicrobial usage, No - No antimicrobial usage, and No - Yes antimicrobial usage |
| *Collinsella* | Unique | No - No antimicrobial usage | *Leucobacter* | Pan | Yes - Antimicrobial usage, No - No antimicrobial usage, and Yes - No antimicrobial usage |
| *Conchiformibius* | Unique | No - No antimicrobial usage |  |  |  |

**Table S7 continued.**

| **Genera** | **Type** | **Group** | **Genera** | **Type** | **Group** |
| --- | --- | --- | --- | --- | --- |
| *Massilia* | Core | Yes - Antimicrobial usage, Yes - No antimicrobial usage, No - No antimicrobial usage, and No - Yes antimicrobial usage | *Romboutsia* | Unique | No - No antimicrobial usage |
| *Microbacterium* | Pan | Yes - Antimicrobial usage, No - No antimicrobial usage, and No - Yes antimicrobial usage | *Rothia* | Unique | No - No antimicrobial usage |
| *Moraxella* | Pan | Yes - Antimicrobial usage, Yes - No antimicrobial usage, and No - No antimicrobial usage | *Serratia* | Unique | Yes - No antimicrobial usage |
| *Pasteurella* | Unique | No - Yes antimicrobial usage | *Staphylococcus* | Pan | Yes - Antimicrobial usage and No - No antimicrobial usage |
| *Peptoclostridium* | Unique | No - No antimicrobial usage | *Streptococcus* | Core | Yes - Antimicrobial usage, Yes - No antimicrobial usage, No - No antimicrobial usage, and No - Yes antimicrobial usage |
| *Psychrobacter* | Core | Yes - Antimicrobial usage, Yes - No antimicrobial usage, No - No antimicrobial usage, and No - Yes antimicrobial usage | Unknown (other genera)^a^ | Core | Yes - Antimicrobial usage, Yes - No antimicrobial usage, No - No antimicrobial usage, and No - Yes antimicrobial usage |
| *Ralstonia* | Pan | Yes - Antimicrobial usage and No - No antimicrobial usage | *Ureaplasma* | Pan | Yes - Antimicrobial usage and No - No antimicrobial usage |

^a^Unknown should be interpreted lightly as it is not known which family or genera these microbes represent.

**Table S8** Core microbiota identifying the core, pan, and unique genera of dogs with and without dermatological conditions related to their nasal carriage status

| **Genera** | **Type** | **Group** | **Genera** | **Type** | **Group** |
| --- | --- | --- | --- | --- | --- |
| [Ruminococcus]_ gnavus_ group | Unique | Yes - Culture negative for *Staphylococcus* spp. | *Cellulomonas* | Pan | Yes - Culture negative for all bacteria, Yes - Culture negative for *Staphylococcus* spp.,  Yes - MSSP, No - MSSP,  No - MRSA, |
| Solirubrobacterales (genera code: 67-14) | Unique | No - Culture negative for *Staphylococcus* spp. | Chloroplast | Unique | No - MSSA |
| *Acidibacter* | Unique | No - Culture negative for *Staphylococcus* spp. | *Cladosporium* | Unique | Yes - Culture negative for all bacteria |
| *Aspergillus* | Pan | No - Culture negative for *Staphylococcus* spp. and  No - MSSP | *Clostridium*_*sensu*_  *stricto*_1 | Pan | Yes - Culture negative for *Staphylococcus* spp.,  Yes - MRSA and  No - Culture negative for all bacteria |
| *Bacillus* | Unique | Yes - MRSA | *Collinsella* | Unique | Yes - MRSA |
| *Bacteroides* | Unique | No - Culture negative for *Staphylococcus* spp. | *Conchiformibius* | Pan | No - MRSP and  No - MRSA |
| *Bergeyella* | Pan | Yes - MRSA and No - MRSA | *Craurococcus- Caldovatus* | Unique | Yes - Culture negative for all bacteria |
| *Blautia* | Pan | Yes - MRSA and  No - Culture negative for *Staphylococcus* spp. | *Elizabethkingia* | Pan | Yes - Culture negative for all bacteria,  Yes - MRSP, Yes - MSSP, Yes - MSSA,  No - Culture negative for all bacteria,  No-MRSP, No - MSSA, No - MR-CoNS,  No - MS-CoNS |
| Cardiobacteriaceae;  Uncultured genera | Pan | Yes - MSSP,  No - MRSA, No - MSSA, No - MR-CoNS, and No - MS-CoNS | *Embryophyta* | Pan | Yes - Culture negative for all bacteria,  No - Culture negative for *Staphylococcus* spp.,  No - MRSP |

**Table S8 continued.**

| **Genera** | **Type** | **Group** | **Genera** | **Type** | **Group** |
| --- | --- | --- | --- | --- | --- |
| *Enterococcus* | Unique | Yes - MRSA | JGI_0000069-P22 | Pan | Yes - Culture negative for all bacteria,  Yes - Culture negative for Staphylococcus spp., Yes - MRSP, Yes - MSSP, Yes - MSSA,  No - Culture negative for *Staphylococcus* spp.,  No - MSSP, No - MRSA, No - MSSA,  No - MR-CoNS and No - MS-CoNS |
| *Escherichia-Shigella* | Unique | No - MS-CoNS | *Klebsiella* | Unique | No - MS-CoNS |
| *Faecalibacterium* | Unique | No - Culture negative for *Staphylococcus* spp. | *Lactobacillus* | Pan | Yes - Culture negative for *Staphylococcus* spp. and  Yes - MRSA |
| *Gracilibacteria* | Pan | Yes-MRSP, Yes – MRSA, Yes – MSSA,  No - Culture negative for all bacteria, No-MRSP, No - MS-CoNS | *Leifsonia* | Pan | Yes - Culture negative for all bacteria,  Yes - Culture negative for *Staphylococcus* spp., Yes - MSSP, Yes - MRSA,  No - Culture negative for *Staphylococcus* spp.,  No - MSSP and No - MRSA |
| *Granulicatella* | Pan | Yes - Culture negative for *Staphylococcus* spp., Yes-MRSP, Yes - MSSP, Yes - MSSA,  No - MRSP, No - MSSA, No - MR-CoNS,  No - MS-CoNS | *Leucobacter* | Pan | Yes - Culture negative for *Staphylococcus* spp.,  Yes - MRSP, Yes - MSSP,  Yes - MSSA,  No - Culture negative for all bacteria,  No - MSSA, No - MR-CoNS, and  No - MS-CoNS |
| *Helcococcus* | Pan | Yes - MSSP,  Yes - MSSA,  No - MS-CoNS | *Massilia* | Pan | All but Yes - MSSA |

**Table S8 continued.**

| **Genera** | **Type** | **Group** | **Genera** | **Type** | **Group** |
| --- | --- | --- | --- | --- | --- |
| *Microbacterium* | Pan | Yes - Culture negative for all bacteria,  Yes - Culture negative for *Staphylococcus* spp.,  Yes - MSSP, Yes – MRSA,  No - Culture negative for all bacteria,  No - Culture negative for *Staphylococcus* spp.,  No - MRSP, No – MSSP,  No - MRSA, and  No - MR-CoNS | *Rothia* | Pan | No - Culture negative for all bacteria,  No - MSSA and No - MR-CoNS |
| *Moraxella* | Core | All | *Serratia* | Unique | No - Culture negative for *Staphylococcus* spp. |
| *Pasteurella* | Unique | No - MS-CoNS | *Staphylococcus* | Pan | Yes - Culture negative for all bacteria,  Yes - MRSP, Yes - MRSA¸  No - Culture negative for *Staphylococcus* spp.,  No - MSSA |
| *Peptoclostridium* | Pan | Yes - Culture negative for *Staphylococcus* spp. and  Yes - MRSA | *Streptococcus* | Pan | All but No-MRSP and No - MR-CoNS |
| *Psychrobacter* | Core | All | Unknown (other genera)^a^ | NA | All |
| *Ralstonia* | Pan | Yes - Culture negative for all bacteria and No - MRSA | *Ureaplasma* | Pan | Yes - Culture negative for all bacteria,  Yes - MRSP, Yes - MSSA, No-MRSP¸  No - MRSA, No - MR-CoNS, No - MS-CoNS |
| *Romboutsia* | Unique | Yes - MRSA |  |  |  |

^a^Unknown should be interpreted lightly as it is not known which family or genera these microbes represent.

- - 1. **Elastic net logistic regression final Model 1 and Model 2 coefficients**

**Table S9** Coefficient values from the elastic net logistic regression Model 1 and Model 2

| **Model 1: Baseline nasal microbiota  samples coefficients values** | | **Model 2: Follow-up nasal microbiota  samples coefficients values** | |
| --- | --- | --- | --- |
| **Predictor variables** | **Coefficients** | **Predictor variables** | **Coefficients** |
| Intercept | -3.08093924 | Intercept | -0.6971613 |
| Sex (females) | 0.24743138 | Antimicrobial usage (yes) | 0.2971285 |
| MRSP | 0.94126839 | Solirubrobacterales (genera code: 67-14) | 6.5813724 |
| *Acidothermus* | 6179.285853 | *Acidibacter* | 11.6946903 |
| *Aspergillus* | -52.2624668 | *Acinetobacter* | 297.3236068 |
| *Bacillus* | -3.46864362 | *Actinobacillus* | -36.7795433 |
| *Cellulomonas* | -24.13939715 | *Afipia* | 4.1678265 |
| *Cutibacterium* | -117.4320037 | *Allorhizobium*-*Neorhizobium*-*Pararhizobium*-*Rhizobium* | -855.5191035 |
| *Defluviitaleaceae*_UCG-011 | 2624.087687 | *Arachnida* | 1023.463391 |
| *Embryophyta* | -4.45976617 | *Bacillus* | 0 |
| *Enterococcus* | -22.19565176 | *Bacteroides* | 0 |
| *Faecalibaculum* | 3130.293505 | *Bergeyella* | -7.7947265 |
| *Gracilibacteria* | 30.70910795 | *Blautia* | 36.9199933 |
| *Helcococcus* | 18.08731161 | *Capnocytophaga* | -98.1121577 |
| JGI_0000069-P22 | -1.26612595 | Chloroplast | 27.5551767 |
| *Lactobacillus* | 0 | *Conchiformibius* | -0.9322229 |
| *Leifsonia* | -38.22020873 | *Corynebacterium* | 0 |
| *Leucobacter* | 9.54634235 | *Faecalibacterium* | 9.684942 |
| *Massilia* | -5.00195322 | *Gracilibacteria* | -15.4126006 |
| *Microbacterium* | -21.24089871 | *Helcococcus* | -23.5108065 |
| *Moraxella* | 0.05358325 | *Leucobacter* | 0 |
| *Neisseria* | 0 | *Microbacterium* | 0 |
| *Nocardioides* | 893.4707945 | *Microvirga* | 0 |
| *Peptococcus* | 1112.915333 | *Neisseria* | 0 |
| *Pseudonocardia* | 186.9289009 | *Porphyromonas* | 0 |
| *Psychrobacter* | -0.4470023 | *Pseudomonas* | -340.5860338 |
| *Rothia* | -59.30697705 | *Psychrobacter* | -0.5219837 |
| *Solirubrobacter* | 0 | *Quadrisphaera* | 1092.426666 |
| *Streptococcus* | -0.32322721 | *Sphingomonas* | 0 |
| *Streptomyces* | 2449.416536 | *Staphylococcus* | -0.126441 |
| Unknown genera | 0.173029 | *Stenotrophomonas* | 110.6762693 |

Using elastic net logistic regression, for the final model for Model 1 (baseline nasal microbiota samples) alpha = 0.1, lambda = 0.07629133, AUC = 1, sensitivity and specificity = 1.

For the final model for Model 2 (follow-up nasal microbiota samples), alpha = 0.2, lambda = 0.412885, AUC = 1, sensitivity and specificity = 1.

**Table S10** Topical antimicrobials used in dogs with and without dermatological conditions

| **Individual dogs’ treatment** | **Topical antimicrobials** | | | |
| --- | --- | --- | --- | --- |
|  | **Topical antimicrobial names** | **Active ingredients** | **Location of application** | **Dosage and duration of use** |
| **Dogs with dermatological conditions** | | | | |
| Dog 4^b^ | Canaural ear drops | 5 mg diethanolamine fusidate,  5 mg framycetin sulfate, 100,000 IU nystatin and  2.5mg prednisolone | Both ears | Applied twice daily for 9 days then switched to Osurnia ear gel. |
|  | Osurnia ear gel | 10 mg florfenicol, 10 mg terbinafine and 1 mg betamethasone acetate per mL | Both ears | Applied twice, 7 days apart. |
| Dog 12^b^ | Flamazine | Silver Sulfadiazine 1% | Left forelimb | Applied twice daily for 5 days. |
| Dog 16^a,b^ | Osurnia ear gel | 10 mg florfenicol, 10 mg terbinafine, 1 mg betamethasone acetate per mL | Both ears | Applied twice, 7 days apart. |
|  | Dermcare Malaseb Shampoo | 20 g/L Chlorhexidine gluconate, 20 g/L Miconazole nitrate | All feet | As required. |
| Dog 35 | Dermotic Ear Ointment | Each mL contains 23 mg miconazole nitrate, 5 mg prednisolone acetate, and 0.696 mg polymyxin B sulphate | Both ears | 0.5 mL applied to both ears once daily for 7 days. |
| Dog 44^b^ | Dermcare Malaseb Shampoo | 20 g/L Chlorhexidine gluconate and 20 g/L Miconazole nitrate | Whole body | Twice weekly. No duration of use mentioned. |
| Dog 46 | Vétoquinol Aurizon ear drops | Each mL contains 3 mg marbofloxacin, 10 mg clotrimazole and 0.9 mg dexamethasone acetate | Both ears | Applied 2 mLs, once daily for 10 days. |
| Dog 63 | Dermotic Ear Ointment | Each mL contains 23 mg miconazole nitrate, 5 mg prednisolone acetate, and 0.696 mg polymyxin B sulphate | Both ears | Applied to both ears, twice daily for 7 days. |
| **Dogs without dermatological conditions** | | | | |
| Dog 40 | Tricin Ear and Eye Ointment | Bacitracin zinc 500 IU/g, Neomycin sulfate 5 mg/g, Polymyxin B sulfate 10,000 IU/g | Right eye | Twice daily for 5 days. |

^a^One sample from Dog 16 was present in ENR Model 1.

^b^One sample each from Dog 4, Dog 12, Dog 16 and two samples from Dog 44 were present in ENR Model 2.

Unless otherwise stated, the names of the manufacturer information were unavailable in the veterinary medical history.

**Table S11** Systemic antimicrobials used in dogs without dermatological conditions

| **Individual dogs’ treatment** | **Systemic antimicrobials** | | |
| --- | --- | --- | --- |
|  | **Antimicrobial name** | **Oral or subcutaneous injection** | **Dosage and duration of use** |
| Dog 5 | Doxycycline | Oral | Once daily 100 mg tablet for 7 days. |
| Dog 19^a^ | Doxycycline | Oral | Twice daily 100 mg tablet for 7 days. |
| Dog 22^a,b^ | Amoxicillin/clavulanate | Oral | Twice daily 500 mg for 14 days. |
| Dog 58 | Amoxicillin/clavulanate | Oral | Twice daily 125 mg for 14 days. |
| Dog 61 | Amoxicillin/clavulanate | Oral | One table 250 mg twice daily for 7 days. |
| Dog 69 | Amoxicillin/clavulanate | Subcutaneous injection | 3 mLs daily. Duration of use unknown (only one sample taken from this dog). |

^a^One sample each from Dog 19 and Dog 22 were present in the ENR Model 1.

^b^One sample from Dog 22 was present in ENR Model 2.

Unless otherwise stated, the names of the manufacturer information were unavailable in the veterinary medical history.

- 1. **Supplementary methods**
     1. **Bacterial isolation of canine nasal methicillin-resistant and -sensitive *Staphylococcus* spp.**

All nasal swabs in Amies medium were initially vortexed (30 seconds), then aseptically snapped into fresh tubes containing Mueller Hinton broth (MH/NaCl; 8 mL; containing 6.5% NaCl) (CM0405B; Thermo Fisher Scientific, Victoria, Australia) with the addition of the respective Amies medium (600 µL). The broths were incubated (37°C; 24 hours) and then the remaining Amies medium per swab (400 µL) was transferred to sterile microcentrifuge tubes and stored (-20°C) for microbiota analysis.

MRSA 2 Brilliance^TM^ agar and Columbia Horse Blood with colistin and nalidixic (CNA) agar plates (PP2475 and PP2032; Thermo Fisher Scientific, Victoria, Australia) were inoculated with the MH/NaCl broths and aerobically incubated (37°C; 48 hours). Colony morphology and catalase tests were used to identify all suspect staphylococcal isolates after incubation. These colonies were sub-cultured onto Sheep Blood Agar (SBA) (PP2133; Thermo Fisher Scientific, Australia) and aerobically incubated (37°C; 24 hours). Subsequent isolates were transferred into 1 mL of brain heart infusion (BHI) (CM1135R; Thermo Fisher Scientific, Victoria, Australia) with 20% glycerol and stored (-80˚C).

- - 1. **MALDI-TOF MS bacterial identification**

Freshly grown overnight colonies were subjected to matrix assisted laser desorption ionisation – time of flight mass spectrometry (MALDI-TOF MS; Bruker Corporation, Bremen, Germany) at the Department of Agriculture and Fisheries Biosecurity Sciences Laboratory, Coopers Plains, Queensland Australia, to identify all suspect nasal staphylococcal isolates. All suspect staphylococcal isolates were pre-treated with 70% formic acid (1 µL) before adding α-cyano-4-hydroxy-cinnamic acid MALDI matrix (1 µL) and following the direct colony transfer method of Timperio, et al. ^3^. Results were interpreted using the standard Bruker interpretative criteria. Isolates with bacterial identification scores between 2.00 to 3.00 were accepted with high-confidence at the species level, while those with 1.70 to 1.99 scores were classified as low-confidence and accepted at the genus level only^4^.

- - 1. **Antimicrobial susceptibility testing**

All staphylococcal isolates were tested using disk diffusion against 13 antimicrobials according to the animal and human Clinical and Laboratory Standards Institute (CLSI) guidelines^5,6^. Oxacillin (OX 1 µg) was used to identify phenotypic MRSP and cefoxitin (FOX 30 µg) was used to identify phenotypic MRSA and methicillin-resistant coagulase-negative *Staphylococcus* spp. isolates. The number of resistant isolates for KF 30 µg (breakpoint for non-susceptibility is ≤28 mm) and CPD 10 µg (breakpoint for non-susceptibility is ≤20 mm) differ as human and animal CLSI guidelines were used respectively to interpret breakpoints. *S*. *aureus* ATCC® 25923 was the quality control organism.

- - 1. **PCR identification of the *mecA* gene in methicillin-resistant *Staphylococcus* spp. isolates**

DNA was extracted from all suspect MRS spp. isolates, following the Chelex DNA extraction methods by Garcha, et al. ^7^, with modifications. Briefly, all isolates were washed in 1 mL sterile water, pelleted, and then resuspended in 6% Chelex matrix (Bio-Rad, Gladesville, New South Wales, Australia). Samples were incubated at 100°C for 8 minutes instead of 95°C, then centrifuged (8,117 x *g*; 10 minutes) and stored at −20°C.

Prior to PCR, DNA was quantified using the NanoDrop^TM^ 1000 Spectrophotometer (Thermo Fisher Scientific, Massachusetts, USA). PCR for the *mecA* gene was conducted on all DNA samples from isolates cultured on MRSA 2 Brilliance^TM^ agar, and for isolates which were phenotypically resistant to oxacillin or cefoxitin. The PCR reaction mixture per sample included: 2 μL DNA, 10 μL Amplitaq Gold 360 Master Mix (Thermo Fisher Scientific), 1 μL of the *mecA* gene (310 bp) forward and reverse primers^8^, 0.2 μL each for the 16S rRNA gene forward and reverse primers targeting the V6-V8 regions^8^, and 5.6 μL ultra-pure water. PCR amplification cycle conditions were denaturation at 95°C for 10 minutes, followed by 10 cycles (94°C, 65–55°C, and 72°C, each for 1 minute), then 25 cycles (94°C, 53°C, and 72°C, each for 1 minute), and a final extension at 72°C for 5 minutes. The positive and negative controls were MRSA ATCC^®^ 43300 and *S*. *aureus* ATCC^®^ 25923, respectively.

PCR products (10 μL) were visualised on a 1.5% agarose gel (containing 1% sodium borate buffer; Bio-Rad Laboratories; and SYBR safe; Invitrogen Australia Pty Limited, Sydney, NSW, Australia), using the GelDoc System (Bio-Rad Laboratories, California, USA). In this study, only isolates that were either resistant to oxacillin or cefoxitin and contained the *mecA* gene, were referred to as methicillin-resistant, while all other isolates are referred to as methicillin-sensitive.

- - 1. **R statistical software packages used in microbiota analysis**

Packages used in R statistical software for the microbiota analysis included:

- Relative abundance: phyloseq^9^, microbiome^10^, dplyr^11^, hrbrthemes^12^, gcookbook^13^, tidyverse^14^.
- Alpha diversity plots: phyloseq^9^, ggplot2^15^, dplyr^11^, ggpubr^16^.
- Beta diversity plots: phyloseq^9^, ggplot2^15^, plyr^17^.
- Core microbiota:
  - Venn Diagrams: phyloseq^9^, microbiome^10^, microbiomeutilities^18^, ggvenn^19^.
  - UpSet plot: rJava^20^, UpSetR^21^, tidyverse^14^, venneuler^22^, grid^23^.
- Co-occurrence networks: phyloseq^9^, igraph^24^, ggplot2^15^.
  - 1. **Data collection**

**Search terms to identify dogs with dermatological conditions**

The search terms used to identify dogs with dermatological conditions (including abbreviations) in the animal shelters database included: dermatitis, interdigital dermatitis, allergic dermatitis, food allergies, fleas, flea allergies, flea allergy dermatitis, contact dermatitis, atopic dermatitis, atopy, atopic, hot spot(s), pruritus, food allergy, cutaneous adverse food reaction, food hypersensitivity reaction superficial pyoderma, pyoderma, deep pyoderma, sarcoptic mange, chronic inflammatory skin disease, chronic dermatitis, excoriation, alopecia, papules, erythema, lichenification, hyperpigmentation, otitis externa, cocci otitis externa, fungal ear infection, and Malassezia.

**Search terms to identify treatment types for dogs with dermatological conditions**

The search terms of commonly used treatment types included: prednisolone, oclacitinib, cyclosporine, lokivetmab, chlorpheniramine, advocate, capstar, dermotic, aurizon ear drops, canaural ear drops, osurnia ear treatment, epiotic, macrolone, malaseb, pyohex, medicated bath(s), cortavance cutaneous spray, and flamazine.

- 1. **Supplementary references**

1 Magiorakos, A. P. *et al.* Multidrug-resistant, extensively drug-resistant and pandrug-resistant bacteria: an international expert proposal for interim standard definitions for acquired resistance. *Clin. Microbiol. Infect.* **18**, 268-281 (2012). <https://doi.org:10.1111/j.1469-0691.2011.03570.x>

2 Lex, A., Gehlenborg, N., Strobelt, H., Vuillemot, R. & Pfister, H. UpSet: Visualization of intersecting sets. *IEEE Trans. Vis. Comput. Graph.* **20**, 1983-1992 (2014). <https://doi.org:10.1109/TVCG.2014.2346248>

3 Timperio, A. M., Gorrasi, S., Zolla, L. & Fenice, M. Evaluation of MALDI-TOF mass spectrometry and MALDI BioTyper in comparison to 16S rDNA sequencing for the identification of bacteria isolated from Arctic sea water. *PLOS ONE* **12**, e0181860 (2017). <https://doi.org:10.1371/journal.pone.0181860>

4 Schulthess, B. *et al.* Identification of Gram-positive cocci by use of Matrix-Assisted Laser Desorption Ionization–Time of Flight Mass Spectrometry: Comparison of different preparation methods and implementation of a practical algorithm for routine diagnostics. *J. Clin. Microbiol.* **51**, 1834 (2013). <https://doi.org:10.1128/JCM.02654-12>

5 Clinical and Laboratory Standards Institute. Performance standards for antimicrobial susceptibility testing; Thirty-second edition CLSI supplement M100. (2022).

6 Clinical and Laboratory Standards Institute. Performance standards for antimicrobial disk and dilution susceptibility tests for bacterial isolated from animals; Fifth edition CLSI supplement VET01S. (2020).

7 Garcha, D. S. *et al.* Changes in prevalence and load of airway bacteria using quantitative PCR in stable and exacerbated COPD. *Thorax* **67**, 1075-1080 (2012). <https://doi.org:10.1136/thoraxjnl-2012-201924>

8 Geha, D. J., Uhl, J. R., Gustaferro, C. A. & Persing, D. H. Multiplex PCR for identification of methicillin-resistant staphylococci in the clinical laboratory. *J. Clin. Microbiol.* **32**, 1768-1772 (1994). <https://doi.org:10.1128/JCM.32.7.1768-1772.1994>

9 McMurdie, P. J. & Holmes, S. phyloseq: An R package for reproducible interactive analysis and graphics of microbiome census data. *PLOS ONE* **8**, e61217 (2013). <https://doi.org:10.1371/journal.pone.0061217>

10 Lahti, L. & Shetty, S. *Tools for microbiome analysis in R*, <<http://microbiome.github.com/microbiome>> (2017-2020).

11 Wickham, H., François, R., Henry, L. & Müller, K. *dplyr: A grammar of data manipulation*, <<https://CRAN.R-project.org/package=dplyr>> (2022).

12 Rudis, B. *hrbrthemes: Additional themes, theme components and utilities for 'ggplot2'*, <<https://CRAN.R-project.org/package=hrbrthemes>> (2020).

13 Chang, W. *gcookbook: Data for "R Graphics Cookbook"*, <<https://CRAN.R-project.org/package=gcookbook>> (2018).

14 Wickham, H. *et al.* Welcome to tidyverse. *The Journal of Open Source Software* **4**, 1-6 (2019). <https://doi.org:https://doi.org/10.21105/joss.01686>

15 Wickham, H. *ggplot2: Elegant graphics for data analysis*. (Springer-Verlag New York, 2016).

16 Kassambara, A. *ggpubr: 'ggplot2' Based publication ready plots*, <<https://CRAN.R-project.org/package=ggpubr>> (2020).

17 Wickham, H. The split-apply-combine strategy for data analysis. *J. Stat. Softw.* **40** (2011). <https://doi.org:10.18637/jss.v040.i01>

18 Shetty, S. A. & Lahti, L. *microbiomeutilities: Utilities for Microbiome Analytics.*, <<https://github.com/microsud/microbiomeutilities/>> (2020).

19 Yan, L. *ggvenn: Draw Venn Diagram by 'ggplot2'*, <<https://CRAN.R-project.org/package=ggvenn>> (2021).

20 Urbanek, S. *rJava: Low-Level R to Java Interface*, <<https://CRAN.R-project.org/package=rJava>> (2021).

21 Gehlenborg, N. *UpSetR: A More Scalable Alternative to Venn and Euler Diagrams for Visualizing Intersecting Sets*, <<https://CRAN.R-project.org/package=UpSetR>> (2019).

22 Wilkinson, L. *venneuler: Venn and Euler Diagrams*, <<https://CRAN.R-project.org/package=venneuler>> (2022).

23 R Core Team. *R: A language and environment for statistical computing*, <<https://www.R-project.org/>> (2022).

24 Csardi, G. & Nepusz, T. The igraph software package for complex network research. *Int. J. Complex Syst.* **1695** (2006).
